# Evidence on Digital HIV Self-Testing From Accuracy to Impact: Updated Systematic Review

**DOI:** 10.2196/63110

**Published:** 2025-03-04

**Authors:** Ashlyn Beecroft, Olivia Vaikla, Nora Engel, Thomas Duchaine, Chen Liang, Nitika Pant Pai

**Affiliations:** 1 Division of Experimental Medicine McGill University Montreal, QC Canada; 2 Research Institute of the McGill University Health Centre Montreal, QC Canada; 3 Athena Institute Faculty of Science Vrije Universiteit Amsterdam Amsterdam The Netherlands; 4 Department of Biochemistry McGill University Montreal, QC Canada; 5 Department of Medicine McGill University Montreal, QC Canada

**Keywords:** digital HIV self-testing, impact, linkage, outcomes, HIV, HIV infection, HIV self-testing, self-testing, digital innovation, systematic review, accuracy, patient-centered, middle- to high-income countries, digital health, mHealth, health education, sexually transmitted diseases, sexual behavior

## Abstract

**Background:**

HIV self-testing has gained momentum following the approval of self-testing methods and novel technological advancements. Digital HIV self-testing involves completing an oral or blood-based HIV self-test with support from a digital innovation.

**Objective:**

We conducted a systematic review on the existing data analyzing digital HIV self-testing accuracy while updating research on digital HIV self-test acceptability, preference, feasibility, and impact.

**Methods:**

We searched Embase and PubMed for records on HIV self-testing with digital support. Included studies significantly incorporated a form of digital innovation throughout the HIV self-test process and reported quantitative data. For accuracy measures, the search spanned January 1, 2013, to October 15, 2024; for patient-centered and impact outcomes, we updated existing literature (June 16, 2021, to October 15, 2024) reported in a previous systematic review. Studies’ quality was assessed using the QUADAS 2 Tool, Newcastle-Ottawa Scale, and Cochrane Risk of Bias Tool 2.

**Results:**

Fifty-five studies (samples ranging 120-21,035, median 1267 participants) were summarized from 19 middle- to high-income countries. Seven studies reported on the accuracy of HIV self-testing with innovations from >5000 participants. Diagnostic performance metrics, including point estimates of specificity, sensitivity, positive predictive value, and negative predictive value were measured (n=3), and ranged from: 96.8% to 99.9%, 92.9% to 100.0%, 76.5% to 99.2%, and 99.2% to 100.0%, respectively. The percentage of invalid test results for oral and blood-based self-tests ranged from 0.2% to 12.7% (n=4). Fifty-one studies reported data on metrics beyond accuracy, including acceptability, preference, feasibility, and impact outcomes from >30,000 participants. Majority (38/51, 74.5%) were observational studies, while 25.5% (13/51) reported data from randomized controlled trials. Acceptability and preference outcomes varied from 64.5% to 99.0% (14/51) and 4.6% to 99.3% (8/51), respectively. Feasibility outcomes included test uptake (30.9% to 98.2%; 28/51), response rate (26.0% to 94.8%; 7/51), and visits to web-based providers (43.0% to 70.7%; n=4). Impact outcomes assessed new infections (0.0% to 25.8%; 31/51), first-time testers (2.0% to 53.0%; 26/51), result return proportions (22.1% to 100.0%; 24/51), linkage to care as both connections to confirmatory testing and counseling (53.0% to 100.0%; 16/51), and referrals for treatment initiation (44.4% to 98.1%; 8/51). The quality of studies varied, though they generally demonstrated low risk of bias.

**Conclusions:**

Digital innovations improved the accuracy of HIV self-test results, and were well-accepted and preferred by participants. Operationally, they were found to be feasible and reported impacting the HIV self-testing process. These findings are in favor of the use of digital HIV self-test innovations as a promising support tool and suggest that digital HIV self-tests’ service delivery models hold promise in not only facilitating HIV testing but also impacting operational outcomes that are crucial to reaching Joint United Nations Program on HIV/AIDS targets in middle- to high-income countries.

**Trial Registration:**

PROSPERO CRD42020205025; https://www.crd.york.ac.uk/prospero/CRD42020205025

## Introduction

### Background

To meet the Joint United Nations Program on HIV and AIDS (UNAIDS) targets, HIV self-testing strategies have been deployed in many countries around the world. HIV self-test methods have risen in popularity since being approved by the Food and Drug Administration (FDA) in 2012, with HIV self-tests now offered as oral or blood-based options, allowing users to receive their results within minutes [1]. Accompanying the rise in HIV self-test usage, digital innovations that support HIV self-testing are becoming widely used in health care, enhancing this method of self-testing. The World Health Organization (WHO) defines digital health innovations as technologies that contribute to improved health outcomes [2]. These technologies can be implemented within any step of the self-testing process including HIV self-testing pre- and posttest counseling, evidence-based knowledge sharing, test ordering, test result interpretation, linkages to care, referrals, and retention in care. Each of these steps plays a major role in the success of HIV self-testing programmatic implementation. The WHO advocates for the adoption and scale-up of digital health innovations to advance global health developments, in support of its One Health Agenda, which includes digital health as the great enabler of the One Health Agenda, which brings light to the integration and unity required to balance the health of humans, animals, and ecosystem [3]. HIV self-test with digital support holds the potential in improving patient-centered and operational outcomes. In the light of recent developments in digital health at the WHO, evidence synthesis was deemed necessary to summarize evidence on digital HIV self-test–based service delivery models [4].

Systematic reviews have sought to summarize global evidence on HIV self-test outcomes, as a result of the increased popularity of self-testing methods. A previous systematic review evaluated studies from January 1, 2010, to June 15, 2021, that focused on patient-reported outcomes including acceptability and preference, and operational feasibility and impact of HIV self-testing methods along with digital innovations [5]. The digital supports examined in this review included web-based interventions (ie, websites, chatbots, and online video counseling), social media and app-based innovations, SMS-based innovations, and digital vending machines. This review found these forms of digital support resulted in reasonably high acceptability (77%-97%), preference (53%-100%), feasibility (93%-95%), and impact (53%-100%) [5]. However, this review neglected to include accuracy metrics, due to insufficient literature at that time.

Despite the expansive integration of digital methods with self-testing during and following the COVID-19 pandemic, there has been limited work focusing on the role of digital support complementing HIV self-testing, particularly regarding the diagnostic performance of these tests. One secondary data analysis of a completed randomized controlled trials quasi–randomized controlled trial (quasi-RCT) has recently evaluated the accuracy metrics of digital HIV self-testing methods and compared them to laboratory HIV reference standards and reported high sensitivity and specificity at 95.5% and 99%, respectively [6]. To inform policy guidance, we need many more studies that analyze the diagnostic performance of digital HIV self-test. A related systematic review evaluating the accuracy of HIV self-testing, notably without digital supports, found that while participants generally performed the self-tests well, errors such as improper kit preparation, misconducting sample collection, and buffer solution spillage were common [7]. The performance accuracy of self-tests also varies tremendously depending on the user, type of test, and test setting, such as whether assistance from health care professionals is available. A systematic review comparing the accuracy of HIV self-tests conducted by the general population to that of diagnostic tests performed by health care practitioners concluded that HIV self-testing innovations were a reliable and accurate method of testing when used by the general population [8]. Although these reviews provide valuable insights into the accuracy of HIV self-testing, no systematic reviews have yet assessed the impact of digital innovations on HIV self-testing accuracy.

Considering the FDA only approved oral-based HIV self-tests in 2012, it is unsurprising that digital innovations in self-testing have rapidly evolved over the past decade, with the COVID-19 pandemic further accelerating advancements in this space, particularly in facilitating accuracy reporting [9]. This updated systematic review aims to address gaps in evidence regarding the effect of digital HIV self-test supports, while providing guidance for policy, practice, and research. These findings are especially relevant in the context of One Health and the global expansion of digital health service delivery models.

### Objective

Our objective was to update global evidence on digital HIV self-test, assessing whether digital supports improved patient outcomes, ameliorated the process of conducting and interpreting self-test results, and affected impact outcomes.

We aimed to update evidence on all outcomes with digital innovations for HIV self-test, including patient-reported and operational outcomes such as acceptability, preference, feasibility, and impact.

## Methods

### Search Strategy

The first search strategy included in this review was conducted as an extension of the earlier systematic review by McGuire et al [5], which reported on the secondary outcomes (acceptability, preference, feasibility, and impact), but excluded accuracy. This review extended the search to include accuracy studies from 2013, reflecting the FDA’s approval of oral-based HIV self-testing in 2012 [9]. The second search strategy was developed to expand upon the same secondary outcomes of McGuire et al [5], but from June 2021 onwards, where the original search strategy ended.

We followed the original protocol, registered on PROSPERO, and modified the strategy to incorporate the newly added accuracy outcome.

We followed the PRISMA (Preferred Reporting Items for Systematic Reviews and Meta-Analyses) guidelines and Cochrane guidelines to conduct and report this review.

No study participants or members of the public were involved in the design, conduct, or reporting of this review.

### Information Sources

Two reviewers (AB and OV) searched 2 electronic databases (PubMed and Embase), first, for citations pertaining to previously unreported accuracy outcomes for the period of January 1, 2013, to October 15, 2024 (Multimedia Appendix 1); and second, for new citations pertaining to secondary outcomes (acceptability, preference, and feasibility) and impact outcomes for the period of June 16, 2021, to October 15, 2024 (Multimedia Appendix 1). There was an overlap of 3 papers that qualified for both the accuracy and patient-centered outcomes [10-12]. No restrictions were placed on language in either search. We retrieved all full-text studies and conference abstracts, with both authors (AB and OV) independently screening publications.

We also searched abstracts for the following conferences: Annual Canadian Conference on HIV and AIDS Research 2021/2022/2023, Infectious Diseases Society of America IDWeek 2021/2022, the 11th International AIDS Society Conference on HIV Science 2021, and the 24th International AIDS Conference (AIDS 2022).

### Eligibility Criteria

We included all studies (observational and interventional [trials and quasi-RCT based experimental] designs) evaluating digital innovations facilitating HIV self-test in any country and those reporting quantitative results.

We included studies only if the digital supports were significantly used in the process of performing HIV self-tests (ie, administration, education, communication, result interpretation, and linkage to care).

We excluded qualitative studies, reviews, protocols, modeling studies, commentaries, narrative studies, case reports, and editorials; studies that did not have HIV as their primary focus did not include HIV self-test or did not use a digital technology; as well as studies not written in English.

### Study Selection and Data Abstraction

Titles, abstracts, and full texts were screened independently by 2 reviewers (AB and OV) for eligibility, and the final included data were independently abstracted.

Abstracted data included study design, country, sample size, study population characteristics, digital innovation type, intervention description, and key findings.

A senior reviewer (NPP) was consulted for the resolution of disagreements.

### Summary Outcome Measures and Narrative Synthesis of Results

To evaluate the integration of digital technology in HIV self-testing, we focused on a primary outcome (accuracy), and secondary outcomes (acceptability, preference, feasibility, and impact).

Accuracy metrics included sensitivity, specificity, positive predictive value (PPV), and negative predictive value (NPV), defined in Table 1 [13].

**Table 1:** Accuracy metrics definitions.

Metric	Definition
Sensitivity	A test’s ability to correctly identify those with the disease, the proportion of true positives (numerator) over the proportion of true positives and false negatives (denominator).
Specificity	A test’s ability to correctly identify those without the disease, the proportion of true negatives (numerator) over the proportion of true negatives and false positives (denominator).
Positive predictive value	The probability that the disease is present, given the test result is positive, the number of true positives (numerator) over the number of true positives and false positives (denominator).
Negative predictive value	The probability that the disease is absent, given the test result is negative, the number of true negatives (numerator) over the number of true negatives and false negatives (denominator).
Invalid test result	A test result that did not correspond to the expected outcome of control and test lines for either negative or positive results.

Furthermore, secondary outcomes (patient-centered acceptability, feasibility, and preference) and operational impact outcomes were adopted from a previously published systematic review and are defined in Table 2 [5].

**Table 2:** Outcome measures and definitions.

Outcome	Definition
Acceptability	The ease of use and willingness of participants to use digital innovations for HIV self-testing, defined as those who agreed to use or try the digital innovation (numerator), over all those who were enrolled in the study (denominator).
Preference	The proportion of study participants who preferred HIV self-tests with digital supports over conventional HIV testing, defined as those who prefer this method of self-testing (numerator) over all those who were enrolled (denominator).
Feasibility	The convenience of using HIV self-test with digital supports, reported with self-test uptake, response rate, and visits to web-based HIV self-testing providers.
Impact	A statistically significant improvement in measured outcomes compared with a comparator group or a net change in outcomes among a particular group that can be attributed to specific intervention–reported metrics include the proportion of first-time testers, detection of new infections, HIV self-test kit return rate, the proportion of participants linked to continued care including counseling or confirmatory testing, and proportion of those referred to treatment.

### Quality Assessment

We assessed the quality of diagnostic accuracy studies with the QUADAS-2 (quality of diagnostic accuracy studies) Tool for accuracy outcome papers [14]. We used the Cochrane Risk of Bias Tool 2 (RoB 2) to assess the quality and potential risk of bias of RCTs [15], and the Newcastle-Ottawa Scale (NOS) was used for cohort and cross-sectional studies [16,17].

## Results

### Study Selection

For the first search, 146 studies were retrieved, 139 were excluded, and there was a final set of 7. For the second search, 543 studies were retrieved, 492 were excluded, and there was a final set of 51. Out of 3 of the studies found overall were used for both primary and secondary outcomes, therefore a total set of 55 studies was included in this review. We found significant heterogeneity in the reporting of populations, study designs, HIV self-test interventions, and outcome metrics that precluded a meta-analysis.

#### Accuracy

**Figure 1:**
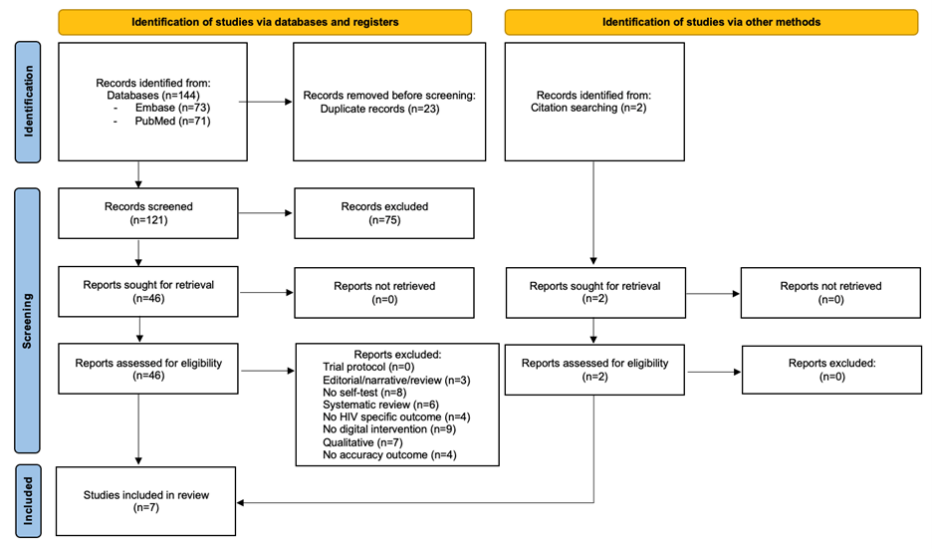
PRISMA (Preferred Reporting Items for Systematic Reviews and Meta-Analyses) flow diagram, accuracy outcome.

For this outcome, 7 studies were included in the final analysis (Figure 1).

#### Secondary Outcomes (Acceptability, Preference, Feasibility, and Impact)

**Figure 2:**
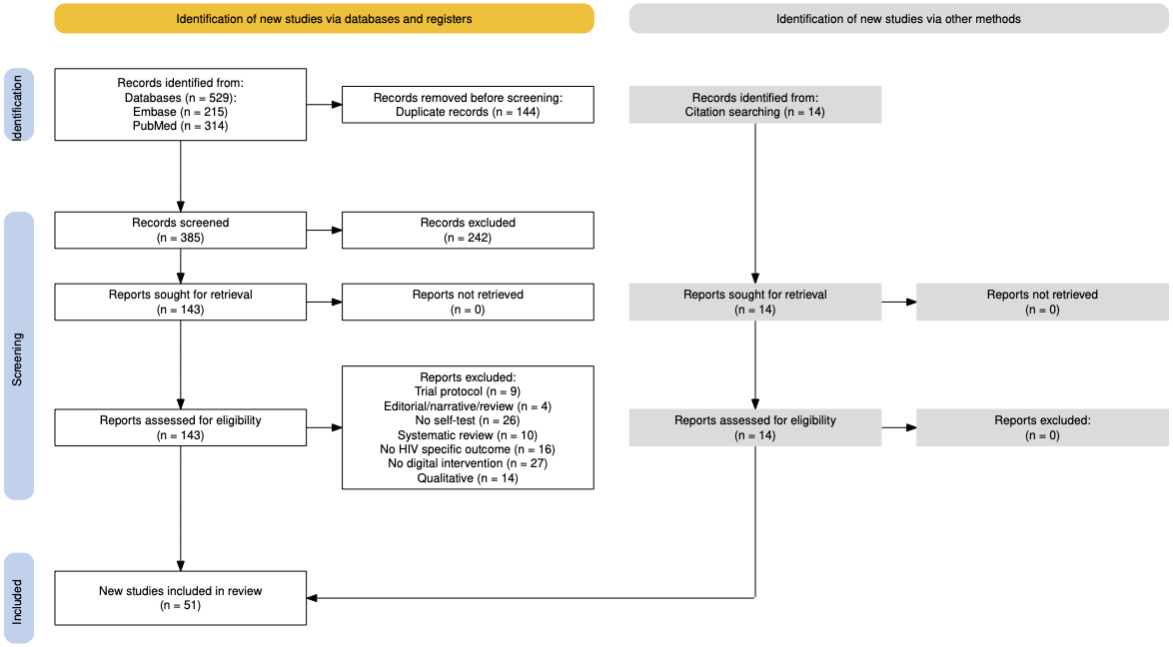
PRISMA (Preferred Reporting Items for Systematic Reviews and Meta-Analyses) flow diagram secondary outcomes.

For these outcomes, 51 studies were included (Figure 2).

### Study Characteristics

#### Accuracy

Accuracy studies were reported from each the following countries: Canada, Kenya, South Africa, the United Kingdom, the United States, and China (n=2). Out of 4 (57.1%) were cross-sectional, while 2 (28.6%) were cohort, and 1 (14.3%) was a quasi-RCT.

Study sample sizes ranged from 271 to 3259 participants.

Study characteristics are tabulated in, Table S1 in Multimedia Appendix 1.

Most studies used website-based HIVST innovations (5/7, 71.4%), one used app-based digital support (1/7, 14.3%), and another used a multimodal approach to HIV self-test including app-based, SMS-based, social media, and web-based innovations (1/7, 14.3%).

Out of 4 (57.1%) studies were conducted in the general population, and the other 3 (42.8%) recruited key populations (men who have sex with men [MSM], or specifically Black, African American, or Latinx MSM).

#### Secondary Outcomes

In total, 51 studies reported on secondary outcomes from studies conducted in 18 countries. These included: Canada (19.6%, 10/51), China (19.6%, 10/51), the United States (13.7%, 7/51), South Africa (11.8%, 6/51), India (7.8%, 4/51), and Thailand (3.9%, 2/51). One study (2.0%, 1/51) was reported in each of the following countries: Australia, Brazil, Japan, Kazakhstan, Malaysia, Nigeria, Philippines, Uganda, Ukraine, the United Kingdom, Vietnam, and Zimbabwe.

Sample sizes varied from 120 to 21,035 (median 1269, IQR 3305) participants (Table S2 in Multimedia Appendix 1).

#### Study design

A majority (74.5%, 38/51) of studies were observational (62.7%, 32/51 cross-sectional; 11.8%, 6/51 cohort) and 25.5% (13/51) of studies were RCTs.

#### Population

Nearly half of the studies (47.1%, 24/51) focused on MSM populations, while the rest (11.8%, 6/51) specifically focused on Black, African American, or Latinx MSM, and 7.8% (4/51) were conducted in transgender women. Over one-third (43.1%, 22/51) of studies were evaluated in the general population. One study only focused on women, and another only focused on Black, Caribbean, and African-Canadian people.

#### Interventions

Over half (56.9%, 29/51) evaluated outcomes from web-based innovations, 21.6% (11/51) evaluated mobile app-based innovations, 5.9% (3/51) evaluated social media, digital vending machines, and SMS were evaluated in one study each (2.0%, 1/51), and 11.8% (6/51) of studies evaluated multimodal interventions (web-based, social media, or SMS-based).

### Risk of Bias in Studies

#### QUADAS 2 Tool

Using the QUADAS 2 Tool, we found a low risk of bias for the studies. 71.4% (5/7) missed details on the reference standard test used.

Key findings can be found in Table S3 in Multimedia Appendix 1.

#### RoB2

Using the RoB 2, we found low risk of bias for the included RCTs. Blinding of participants was reported by 30.8% (4/13) of studies. Although blinding of the participants and assessors through the trial and analysis was not possible due to the assessed outcomes of digital supports, there were no reports of deviations from the intended interventions that arose in any of the studies. As well, every study had majority or all the data present in the analysis, and attrition bias was minimized. Finally, the outcome was reported in detail, minimizing the possibility of reporting bias.

Further details regarding these key findings can be found in Table S4 in Multimedia Appendix 1.

#### NOS for Cross-Sectional Studies

Using the NOS, we found that cross-sectional studies were generally fair. 53.1% (17/32) of these studies had an overall fair quality. The possibility of selection bias, confounding, or outcome and exposure misclassification was found in 18.8% (6/32), 25.0% (8/32), and 34.3% (11/32) of these studies, respectively. Many of the cross-sectional studies (90.6%, 29/32) had unjustified sample sizes (no sample size calculation was mentioned).

Further details regarding these key findings can be found in Table S5 in Multimedia Appendix 1.

#### NOS for Cohort Studies

Using the NOS, we found cohort studies were of average quality. Possibility of selection and confounding bias were low at 33.3% (2/6), respectively. However, 83.3% (5/6) of the cohort studies had a risk of attrition bias.

Further details regarding these key findings can be found in Table S6 in Multimedia Appendix 1.

### Primary Outcome: Accuracy

A total of 7 studies evaluated the accuracy of HIV self-test integrated with the use of digital innovations [6,10-12,18-20]. Overall, 42.3% (3/7) reported on blood-based self-tests [12,18,20], 42.3% (3/7) on oral-based self-tests [6,10,19], and one allowed participants to choose between oral or blood-based self-tests but reported no differences in accuracy between the 2 types of tests [11].

A trend was noted on the reporting of invalid test results, since a majority (57.1%, 4/7) of studies reported on the percentage of invalid test results. Invalid tests reported ranged from <0.2% to 12.7%, for both blood- and oral-based self-tests [10-12,18].

One study in which the participants were asked to perform the self-test on site and report their result to the researcher as soon as it was available, reported on diagnostic performance metrics (ie, sensitivity, specificity, PPV, and NPV) of the oral-based Aware HIV self-testing, but notably only as point estimates without CI: Sn 92.9% (13/14), Sp 96.8% (121/125), PPV 76.5% (13/17), and NPV 99.2% (121/122) [19].

A study that was a secondary data analysis of a quasi-RCT found high accuracy metrics when comparing the HIV self-testing result with the reference standard of 2 rapid tests and 1 HIV RNA test [6]. Sensitivity was reported as 95.52% (95% CI 94.48%-96.56%), specificity was 99.93% (95% CI 99.79%-100.00%), PPV was 99.22% (95% CI 98.78%-99.67%), and NPV was 99.57% (95% CI 99.24%-99.90%) [6].

Another notable study that reported on such metrics did so using an AI-based program that was able to read and interpret the result of the participants’ self-test [20]. In this study, sensitivity was 100% (44/44), as well as NPV. Specificity was also high at 98.8% (798/808), but the PPV was lower at 81.5% [20].

A fourth study allowed participants to choose between an oral- or blood-based HIV self-testing, but did not include mention of the manufacturers of these tests in the publication. Only 2 of the 4 positive HIV self-tests were confirmed to be positive but neglected to mention if these results were from the oral- or blood-based self-testing options. The participants of this study ordered the self-test and completed it at home, then uploaded their self-test results onto the web-based platform, which could have contributed to the few positive self-tests received. This study also reported the agreement of test interpretation between participants and the research team as 99.1% (95% CI 97.4%-99.8%) [11].

A study from the United Kingdom reported on 24,717 blood-based self-test kits purchased during the study, of which there was only a <0.2% rate of invalid tests and only 3 false positives were reported, which was much lower than they had expected [18].

Further details regarding these key findings can be found in Table S7 in Multimedia Appendix 1.

### Secondary Outcomes

Outcome measures beyond accuracy become important when real life implementation is called into question. Due to this, we explored patient-reported acceptability and preference, and operational feasibility and impact outcomes.

Further details regarding these key findings can be found in Table S8 in Multimedia Appendix 1.

#### Acceptability

Overall, 27.5% (14/51) of the studies reported acceptability measures [21-34].

#### Ease of Use

A total of 8 studies (57.1%, 8/14) reported acceptability as the ease of use of the digital interventions [22,23,28-30,32-34]. In 1 study, 64.5% of participants found the digital innovation easy or very easy to use [29]. Comparatively, an RCT evaluating an infographic for HIV self-testing found that 73.5% of participants agreed that it was easy to use [30]. Another study reported that only 7.5% (9/120) of participants said the digital support was easy to use, though one participant mentioned: “*It is very easy to use, less stressful very understandable, it is the best and very advanced product one could ever wish for”* [28]. Alternatively, 1 study measured ease of use using a 5-point Likert scale and found a score of 3.8 (SD 1.6) for the ease of uploading results, and a score of 4.2 (SD 0.9) for ease of finding a clinic using the digital innovation [23].

#### Willingness to Use

Overall, 42.8% (6/14) reported on the willingness of participants to use the self-test with the digital intervention, which was consistently high (72.2%-99.0%) [23-27,31]. A quasi-RCT in the Philippines demonstrated the willingness of participants to use a community-based HIV self-test distribution model and found that 99.0% (4163/4205) of respondents were interested in getting an HIV self-test [31].

#### Utility of the Intervention for an Indication

In one study, participants found the digital innovation helpful in reporting of testing results (97.6%), the concept of window period (88.8%), understanding their current risk (85.6%), reducing high-risk behaviors (80.0%), and mitigating fear of HIV testing (72.0%) [22].

#### Preference

Preference was assessed in 15.7% (8/51) of the studies [11,24,28,35-39].

Preference for the digital innovation versus standard testing methods ranged from 63.0% to 99.3% (3/51) [24,28,37]. An outlier study found that only 5.9% (128/2181) of participants chose to do an HIV self-test with the support of an application on their phone, instead of HIV testing conducted by a health care provider [39].

One study analyzed data from 794 participants on their reasoning for preference of HIV self-testing online versus other methods of testing. These were reportedly: convenience (726/794, 79.0%), not wanting to wait for results (402/794, 44.0%), not wanting to talk about sex with anyone (298/794, 33.0%), not having time to go elsewhere for testing (268/794, 29.0%), and fear of stigma (205/794, 22.0%) [35]. Notably, 21.0% (40/190) of participants who responded to the survey question said they would not test elsewhere at all [35].

One study reported on lower rates of preference; a majority of participants who requested a self-test did not want any form of support (338/434, 77.9%) [11]. However, of those who did want support, digital forms of support including instant messaging, video calls, and chatbots were preferred by 65.8% (102/155) of respondents, compared to in-person support (47.7%, 74/155) [11].

#### Feasibility

Feasibility was assessed in 60.8% (31/51) of the studies [10-12,21-23,29,31,34,35,40-60].

#### Uptake

Overall, 90.3% (28/31) of these studies reported uptake of self-tests [10-12,22,31,34,35,40-60], which varied across studies (30.9%-98.2%; 11/31) [10,11,22,31,40-42,44,45,48,49,54]. Out of 9 of the remaining studies measured uptake as the number of HIV self-test kits ordered throughout the study. This included 701 kits ordered by 604 participants, 834 tests ordered by 309 participants, 5840 kits ordered by 5324 participants, 5235 kits ordered by 3627 participants, 5323 kits ordered by 4859 participants, 13,334 kits ordered by 11,332 participants, and 3 studies in which each participant ordered a single test (2610, 7315, and 21,035) [12,46,47,50,52,53,55,57,58]. An Australian study reported that of the 794 participants who ordered an HIV self-testing, 95 of them ordered multiple self-testing kits, between 2 to 7 kits per person [35]. An RCT conducted in the United States investigated the effect that a peer-led online community had on the uptake of the blood-based myLAB Box HIV self-testing [51]. This study found there was an increase of 6.3% in test uptake between the intervention and control groups, with an odd ratio (OR) of 1.43 (95% CI 1.04-1.95, *P*=.03) [51]. Another RCT conducted in China analyzed the effect of monetary incentives and online peer referrals on test uptake of the blood-based SD BIOLINE HIV and syphilis self-test [52]. This study found that the 102 control participants ordered 222 kits, the 103 participants of the monetary intervention group ordered 275 kits, and the 104 participants in the monetary and peer referral intervention group ordered 337, all of which were ordered using a digital intervention [52].

#### Proportion of Participants Who Responded to the Intervention

About 22.6% (7/31) of studies reported how many of the participating people actually responded to the intervention, with one study reporting a response rate of 26.0% (2467/9505), and the others 61.1%-94.1% (216/345 to 96/102) [11,29,35,47,51,52,54]. The outlying response frequency of 26.0% 2467/9505 referred to responses of an optional online survey asking questions on participants’ quality of experience, which was offered once the self-testing process was complete [29]. In RCT, the response proportion was higher among the intervention group (93.4%, 421/450) compared with the control group (92.9%, 418/450) [51]. As well, another RCT found the response rate to be higher among the intervention groups (94.2% and 96.2%) compared with the control group (94.1%) [52].

#### Visits to the Web-Based Provider

Out of 4 studies reported such, of which three reported as percentages of 43.0% (1475/3431) of participants interacted with the content, 70.7% (531/751) of participants logged onto the app, and 67.9% (19/28) clicked on messages sent on the app [21,23,60]. The remaining paper reported that the study website was viewed about 266,000 times [47].

#### Impact

Impact was measured in 88.2% (45/51) of the studies [4,10-12,21-25,27,29,31-35,37-50,52,53,55-67].

#### Detection of New HIV Infections

This was the most prominent metric used in 68.9% (31/45) of the studies [4,10,11,22-24,29,31-33,35,40-50,52,55-59,61,62, 64]. A majority of these papers (61.3%, 19/31) reported the proportion of new infections by HIV self-tests result which varied from 0.0% to 25.8%, which were confirmed by laboratory result [10,11,23,24,29,31,32,40,41,44-46,48,49,57-59,61,64]. One study reported that 18 participants returned a positive self-test result [61]. Another study found that of 5048 returned self-test results, 165 were positive [47]. One study noted that 41.1% (130/314) of the participants that tested positive had not received a positive result before [29]. The other 8 papers reported the proportion of new infections as those confirmed by laboratory testing, ranging from 0.0% to 10.1% [4,22,33,35,42,43,52,56]. One study did not specify how many results were returned, but stated that 86 people who conducted the self-test were confirmed via laboratory result at the project sites [50]. Another study found that out of those who sought confirmatory testing, 96.8% (522/539) were confirmed to be HIV positive [55]. A quasi-RCT found the proportion of new infections to be higher in the intervention group (8.9%), which included unsupervised and supervised self-testing, compared with the control group (6.8%) risk ratio (RR; 1.304, 95% CI 1.023-1.665) [4].

#### The Proportion of “First-Time Testers”

Reported in 57.8% (26/45) of the studies [11,12,21,24,25,27,31,34,35,37,42,44,45,48,49,52,53,55-57, 61-64,66,67]. The proportion of first-time testers for 23 of these studies ranged from 2.0%-53.0% [11,12,21,24,25,27,31,34,35,37,42,44,45,48,52,53,55-57, 62,64,66,67]. In addition, one study looked at 1070 returned self-tests and found 43 of them to be positive, all of which were from first-time testers [49]. One study compared the proportion of first-time testers between age groups and found that 53.0% of those who were 18-24 years old were first-time testers, compared with only 20%-30% of those in the higher age groups (OR 3.30, 95% CI 2.88-3.78) [63]. Another study looked at the difference in rate of first-time testers among White participants compared with African, Caribbean, and Black participants and found that the proportion of White first-time testers was 21.0%, compared with 30.0% among African, Caribbean, and Black participants [61].

#### Result Return Proportion

Defined as the percentage of reported results from participants and was captured in 53.3% (24/45) of the studies, ranging from 22.4%-100.0% [10,12,23,29,31,32,38,39,41,44-46,48,49,52,53, 55,56,60-62,64,65,67]. An RCT found that the return rate among the control group (94.0%) was higher than the intervention group that did not receive online peer referral (93.4%), but was lower than the intervention group that did receive online peer-referral (97.3%) [52].

#### Linkage to Care

Observed in 46.6% (21/45) of studies [4,22,24,29,31,33-35,40,42,43,45-49,53,55,57,62,64]. 76.2% (16/21) of these studies reported this metric as confirmatory HIV testing and linkage to treatment which varied from 53.0%-100.0% [22,24,29,31,33-35,40,42,45,47,53,55,57,62]. Out of 8 studies (38.0%) reported linkage to care as the percent of confirmed HIV-positive participants who were referred to and initiated antiretroviral therapy (ART) and ranged from 44.4%-98.1% [4,43,46,48,49,53,55,64].

A quasi-RCT reported the highest linkage to care, with a greater proportion for the intervention (99.8%) compared to the control (98.5%; RR 1.012, 95% CI 1.005-1.018) [4]. As well, 98.1% of all those who used HIV self-test were either referred to start ART if they were HIV-positive, or for preventive treatment if they were HIV-negative [4].

#### Referrals to Self-Test

A quasi-RCT also showed that 16.7% of participants in the self-testing group referred someone in their social network, whereas only 3.1% of participants in the conventional testing arm did (RR 5.435, 95% CI 4.024-7.340) [4]. In another RCT, it was reported that 78.0% (479/618) of participants had given a self-test to a social network associate [67].

## Discussion

### Principal Findings

#### Accuracy

Invalid tests were not explicitly defined in any of the studies but were assumed to refer to a result that was neither negative nor positive. Invalid results are a product of a defective test, incorrect testing conduct, or result in misinterpretation. Invalids are an important factor to consider for diagnostic performance, as users’ trust in the self-testing process may diminish when an invalid result is received. The WHO and other governing bodies lack defined thresholds for acceptable invalid results in screening. We found the highest invalid rate (12.7%) occurred in Canada, using the blood-based bioLytical INSTI self-test, Canada’s sole approved HIV self-testing so far [12]. There is a need to define an acceptable threshold of invalid tests at the level of approval.

Sensitivity, specificity, PPV, and NPV are crucial for evaluating the performance of HIV self-testing and determining the reliability of results. Although 7 studies reported high diagnostic performance with HIV self-testing as the low proportion of invalid tests or high sensitivity, specificity, PPV, and NPV, the overall body of literature in this area remains sparse.

One study analyzed the impact of a digital reader in self-result interpretation, compared to the participants’ interpretation [20]; however, no studies assessed the improved accuracy of digital intervention-enhanced HIV self-tests compared to those without digital interventions that are widely accessible to the general population. This highlights the need to explore the potential of digital innovations in improving the accuracy of HIV self-test.

Furthermore, it is important to highlight that one of the studies reporting all the ideal accuracy measures evaluated the impact of internet use on HIV self-test uptake and performance, not the direct influence of digital interventions on the self-testing process [19]. This indicates a research gap in understanding the direct impact of digital interventions on the accuracy of HIV self-testing. This study also reported moderate measures of accuracy, suggesting room for improvement of digital supports that are implemented during the testing process.

One of the other studies that reported the ideal accuracy metrics did so comparing interpretation by (1) participants, (2) pharmacy provider, (3) AI tool, and (4) expert panel of 3 HIV self-test readers [20]. The reported accuracy metrics were comparing the AI tool to the expert panel, instead of between the AI tool and participants, which again highlights this gap in existing research [20].

The final study that reported metrics on diagnostic performance did so comparing the HIV self-test result to the reference standard, reporting high accuracy measurements for each outcome [6]. However, there is still a gap in research comparing the results of HIV self-testing with the support of a digital intervention compared to an HIV self-test result on its own. Future research should aim to analyze the accuracy between HIV self-tests alone versus with digital support, to properly evaluate the direct impact digital innovations have on the self-testing process.

#### Acceptability

Acceptability of digital interventions for HIV self-test varied across studies, indicating that acceptability is influenced by diverse factors. Persistent willingness to use digital interventions across studies (72.2%-99%) suggests openness and receptiveness to incorporating digital technologies into the self-testing process. To improve the acceptability of digital interventions, future developments should consider comprehensive HIV self-testing integration, including sample collection and result interpretation.

Ease of use varied significantly in definition across the 8 studies that reported this metric, but overall, most study participants found the digital innovations easy to use. It is important to note that the demographics of the participants in these studies may play a role in the reported ease of use. For example, in 1 paper, 122/350 participants were people who had used an online HIV self-testing platform before, which may have increased their report of ease of use [22].

#### Preference

This systematic review demonstrates generally high rates of preference (63.0%-99.3%) for digital interventions in the context of HIV self-testing, suggesting notable favorability toward digital innovations for HIV self-test.

One study reported lower rates of preference for any form of support during the HIV self-test process, as most participants in that study expressed a preference for not receiving any support [11]. For those seeking support, digital options were preferred over in-person support, underscoring diverse preferences and the need for tailored interventions. The novelty of digital innovations can be overwhelming, particularly among older generations and individuals who are less familiar with technology, which may influence their preferences of digital interventions for HIV self-test. It is crucial to consider the target population and their digital literacy level, and to provide adequate support, education, and user-friendly interfaces to address potential barriers related to technology adoption.

Another study reporting low preference had given participants the choice between HIV self-testing with a mobile app and HIV testing conducted by a health care provider [39]. This study was conducted among youth aged 16-24 years in Zimbabwe and qualitatively asked the participants why they may have chosen standard HIV testing compared with self-testing with the digital app [39]. Common explanations included low general and digital literacy, limited self-efficacy or agency, fear of testing and need for provider support, lack of private digital or physical space, and technical issues [39]. Given the specific geographic region of the age group included in this study, it is possible that these findings may not be replicated if repeated in another area of the world, for example, the United States, where digital literacy is likely to be improved. However, these qualities still need to be addressed in HIV self-testing and provide people with comfort and a sense of autonomy when self-testing, instead of fear and hesitation. As well, the preference rate found in this study cannot be attributed solely to the digital aspect of self-testing, but the self-testing process as a whole. Increasing awareness of HIV self-testing methods could enhance the outcomes of similar studies, as participants with greater familiarity and understanding of the testing approach may feel more confident in selecting the digital self-testing option.

#### Feasibility

High uptake of self-tests facilitated by digital interventions suggests the broad accessibility and convenience associated with digital HIV self-test.

In one RCT, the overall uptake of HIV self-testing was relatively low, but the inclusion of a digital component increased test uptake by 6% (OR 1.43; 95% CI 1.04-1.95), highlighting the potential of digital interventions to positively influence self-test uptake rates [51].

Another study reported a 25.0% increase in clinical HIV testing through the implementation of a digital solution [43]. This suggests that the widespread application of digital innovations in HIV self-test could contribute significantly to achieving the UNAIDS 2025 targets of increased HIV serostatus knowledge and treatment.

Response rates substantially varied across studies, which may have been influenced by follow-up procedure disparities and the recruitment of vastly diverse populations. Tracking and analyzing response rates are vital to assess the performance of digital interventions and identify effective methods for obtaining feedback via digital means.

Visits to web-based providers varied across the studies, potentially attributed to differences in the methods of exposure to digital innovations. For example, in one study, the intervention involved a campaign about HIV self-test disseminated through social media platforms, which have algorithmic influence on exposure, limiting exposure of certain individuals to the digital intervention [21].

Altogether, the inclusion of digital components has been shown to increase test uptake, and the feasibility of digital interventions are supported by the available evidence. However, response rates and variations in exposure methods should be carefully examined and addressed to optimize the performance and impact of digital interventions in HIV self-testing.

#### Impact

The use of HIV self-testing methods with digital support has shown potential in reaching the first of the UNAIDS 95-95-95 targets, supported by the proportions of new infections and first-time testers.

The parity between new infections identified via HIV self-test and confirmed HIV cases, 9.8% versus 10.1%, indicates that HIV self-test with digital support is a valuable tool for identifying new cases of HIV. Importantly, a study in South Africa reported an increase in the proportion of new infections among the HIV self-testing arm compared to the conventional testing arm (RR 1.305, 95% CI 1.023-1.665), supporting digital aids in enhancing HIV detection [4].

The proportion of first-time testers supports digital innovations’ potential in streamlining HIV self-testing access. Accessibility of digital HIV self-testing methods to the general population is likely to elevate first-time tester rates, capturing those not engaged via traditional methods, and thus expanding testing coverage.

Digital support facilitated high test result returns across studies, likely attributed to the convenience and accessibility digital reporting affords. The ease of reporting results digitally eliminates the need for physical clinic visits, fostering convenience and potentially encouraging higher rates of result reporting.

While one study observed a lower self-test return rate due to mobile-app user attrition, this could be context specific, as the study was conducted in South Africa in 2018/2019, and thus subject to change due to evolving mobile-app utilization trends and self-testing methods used through the COVID-19 pandemic [23].

Linkage to care is a critical aspect of HIV testing. HIV self-testing without digital support have struggled with adequate linkage to care [68]. Digital innovations significantly aid this process, enabling direct participant connections to care without having to navigate the resources themselves. In one study, there was linkage to post-test counselors, who facilitated staging the disease of those who tested positive and assisted in preventative practices for those who tested negative, yielding a robust linkage proportion (99.7%) among the self-testing arm [4].

The findings of this review support the high linkage to care, as the high proportion of individuals who received a positive self-test result were connected to confirmatory testing, underscoring digital support’s role in facilitating real-world follow-up. Furthermore, there was a high proportion of individuals who were confirmed to be HIV-positive and started ART treatment which demonstrates the promising potential of digital interventions in successfully connecting HIV-positive patients to continuative care. These findings highlight the practicality and convenience of digital interventions in the HIV testing and care continuum.

Overall, the use of digital innovations in HIV self-test has resulted in the identification of new infections of HIV, an increased proportion of first-time testers, and a high return rate of test results.

#### Quality Assessment

The accuracy studies generally had low risk of bias; however, a lot of information was not specified. For 57.1% (4/7) of the studies, invalid test results were used as a proxy for real-world performance and were limited by a lack of reference standards to complete the test performance. The studies did not mention if the participants also received confirmatory laboratory testing, except for 2 [6,19]. The RCTs included in this review had a low risk of bias. There is the confidence that these studies were well conducted and have trustworthy results.

Overall, the cross-sectional studies had low risk of confounding bias, but some or high risk of selection bias and outcome misclassification. The high risk of selection bias was mostly due to lack of justified sample sizes. As for outcome misclassification, the assessment of the outcome was consistently self-reported, considering the nature of self-testing, thus increasing the risk of bias. It is also important to note that many of the conference abstracts neglected to include details pertaining to the quality assessment; therefore, the study may have conducted higher quality of research but was not outlined in the abstract alone.

The cohort studies were of average quality. Out of 2 studies had missing data, and another had only one star and were therefore classified to be of low-level comparability [48,57,58]. Overall, the quality of these studies can be trusted in terms of the selection processes, but there should be some hesitation regarding comparability and outcome biases.

### Limitations

The heterogeneity of interventions, outcomes, populations, and settings among the included studies introduced variability and made it challenging to draw definitive conclusions or generalize the findings across various contexts. Heterogeneity in reporting outcomes poses a challenge in synthesizing the evidence. Variation in outcome measures and their assessment methods makes it difficult to pool data and establish a comprehensive understanding of the effectiveness of digital supports. Future studies should adopt standardized outcome measures and reporting guidelines to facilitate meaningful comparisons and meta-analyses.

Limited evidence was available from low-income countries. Many studies included in this review were conducted in the middle- or high-income settings, such as China, the United States, Canada, and South Africa, which may limit the generalizability of the findings to low-income countries. This highlights the need for more research in diverse geographic and socioeconomic contexts to assess the feasibility and effectiveness of digital health interventions across different resource settings.

Several limitations in the review process should also be acknowledged. The search strategy was restricted to 2 databases, which may have resulted in the potential omission of relevant studies published in other databases. Moreover, this review focused on a majority of observational studies, which inherently lack the randomization and control provided by RCTs, potentially influencing the observed effects of digital health interventions.

### Implications and Future Research

This review incorporates 14 RCTs (including 6 quasi-experimental trials), 34 cross-sectional, and 7 cohort studies, providing a comprehensive overview of the existing evidence. These studies offer valuable insights into the effectiveness and potential benefits of digital innovation in health care practices in terms of accuracy, acceptability, preference, feasibility, and impact. Moving forward there is a need to enforce reporting of trials in HIV self-testing as per the CONSORT-EHEALTH (Consolidated Standards of Reporting Trials of Electronic and Mobile Health Applications and Online Telehealth) checklist [69], as well as a need to appreciate the use of quasi-randomized designs that provide a greater richness to the breadth of literature by virtue of implementing these solutions in real-life settings. Single-arm interventional studies also provide evidence on why and how an intervention could be successful in a setting. Bias is an inevitable consequence of all study designs, less so with an RCT, followed by quasi-randomized and single-arm interventional studies, in that order. However, evidence generation with the use of a stronger methodology adds to the evidence base needed to formulate guidelines and to the GRADE (Grading of Recommendation Assessment, Development and Evaluation) approach [70].

In addition, it is important to consider patient preferences in testing processes, as this can have a profound impact on retention rates. Qualitative research underscores digital strategies’ flexibility, allowing individuals to tailor their testing experiences to fit their unique needs and circumstances, and patient satisfaction [71]. For instance, digital HIV self-testing strategies have been shown to mitigate barriers such as stigma associated with in-person testing, while providing users with control over the testing process, including the option to involve, or exclude, others during testing [71-73]. However, challenges remain in ensuring that these tools are accessible to all. These findings call for future research to optimize HIV self-testing methods.

While this review highlights the potential of digital innovations to increase accuracy rates, robust studies are required to comprehensively understand the potential benefits and limitations of digital innovations in enhancing the accuracy of HIV self-testing.

HIV self-testing with digital tools is the next step in the armamentarium in our fight against HIV. These tools are best suited when certain conditions are met, including the following:

1. Digital connectivity and tools to avail it are established and are cheap or relatively affordable, depending on the context and the country setting.
2. Populations using these solutions are digitally literate and health literate and are able to navigate care using these solutions.
3. The pathways to clinical care are made navigable and are often facilitated by a corpus of healthcare providers who can offer assistance to those who call, chat, and engage with them.
4. Dashboards and platforms that are set up are often interoperable and can work with country-level network architecture of electronic records and telemedicine that are in place.
5. Linkage services can be set up easily with these solutions and populations identified with infections can be seamlessly integrated within care pathways.
6. Countries have signed on to the treaty on digital health and are aligned with WHO’s vision of One Health.
7. Linkage to treatment and retention in care are essential for these tools to be optimally effective, not just screening.
8. A corpus of health care counselors and testers have been trained in the use of digital solutions and through training, have become well-equipped to identify the challenges of navigating care pathways and are ready to help task shift to decrease the pain experience in navigating linkage to care and counseling by testers.

### Conclusion

Digital supports enhance HIV self-testing across multiple domains, including improving test accuracy or self-test interpretation and achieving high metrics of acceptability, preference, feasibility, and impact. They have the potential to improve test accuracy by providing clear instructions, result interpretation, and data collection mechanisms, promoting reliable and trustworthy results; however, further evidence is needed in this space.

Digital interventions positively impact acceptability and preference due to their convenience, privacy, and autonomy. Furthermore, they improve the feasibility of HIV self-test, by leveraging technology to provide easy access to testing and result reporting, eliminating the need for physical visits to testing centers or clinics, especially benefitting individuals with time constraints, limited mobility, or those residing in remote areas. Finally, digital supports elevate impact by increasing result returns, attracting first-time testers, identifying new infections, and aiding linkages to care. Leveraging technology’s widespread use, digital interventions extend HIV self-test reach, bridging testing coverage gaps, though more research and implementation data are needed in low-income settings and among key marginalized populations affected by HIV. These populations and settings traditionally lack access to connectivity tools, which may impact equity and access to services. Despite challenges, integrating digital tools aligns with health care’s evolution, offering the potential to revolutionize HIV testing, ultimately leading to better health outcomes for individuals and communities. These interventions bode well to help us achieve UNAIDS 95-95-95 targets, especially for high-risk HIV populations residing in 191 middle- to high-income countries, who constitute a majority of the world’s population.

## Appendix

Multimedia Appendix 1 Extended information.

Multimedia Appendix 2 PRISMA Checklist.

## Abbreviations

ART: anti-retroviral therapy
CONSORT-EHEALTH: Consolidated Standards of Reporting Trials of Electronic and Mobile Health Applications and Online Telehealth
FDA: Food and Drug Administration
GRADE: Grading of Recommendation Assessment, Development and Evaluation
MSM: men who have sex with men
NOS: Newcastle-Ottawa Scale
NPV: negative predictive value
OR: odds ratio
PPV: positive predictive value
PRISMA: Preferred Reporting Items for Systematic Reviews and Meta-Analyses
QUADAS-2: quality of diagnostic accuracy studies
RCT: randomized controlled trials
RoB2: Cochrane Risk of Bias Tool 2
RR: risk ratio
UNAIDS: Joint United Nations Program on HIV/AIDS
WHO: World Health Organization

## Data Availability

All data generated or analyzed during this study are included in this published article (and Multimedia Appendices 1 and 2).
