# Supplementary material for: Evidence on Digital HIV Self-Testing From Accuracy to Impact: Updated Systematic Review
Source: J Med Internet Res. 2025 Mar 4;27:e63110. doi: 10.2196/63110 (PMC11920657; doi:10.2196/63110)
Supplement: Multimedia Appendix 1 [file jmir_v27i1e63110_app1.docx]

**Appendix**

Search String 1: *((hiv[Text Word] OR human immunodeficiency virus[Text Word]) AND (self-test[Text Word] OR self test[Text Word] OR self-testing[Text Word] OR self testing[Text Word] OR self-sample[Text Word] OR self sample[Text Word] OR self-sampling[Text Word] OR self sampling[Text Word]) AND (digital[Text Word] OR Mhealth[Text Word] OR mobile health[Text Word] OR online[Text Word] OR web-based[Text Word] OR website[Text Word]) AND (accuracy[Text Word] OR accurate[Text Word] OR validity[Text Word] OR valid[Text Word] OR sensitivity[Text Word] OR sensitive[Text Word] OR specificity[Text Word] OR specific[Text Word] OR positive predictive value[Text Word] OR ppv[Text Word] OR negative predictive value[Text Word] OR npv[Text Word])).*

Search String 2: *((hiv[Text Word]) AND (self-testing[Text Word] OR self testing[Text Word] OR self-sampling[Text Word] OR self sampling[Text Word]) AND (Mhealth[Text Word] OR mobile health[Text Word] OR digital[Text Word] OR online[Text Word] OR web[Text Word])).*

Table S1: Study characteristics of accuracy studies

| **Reference** | **Study Design** | **Country** | **Sample Size** | **Population** | **Digital Innovation** | **Intervention Description** |
| --- | --- | --- | --- | --- | --- | --- |
| Beecroft et al., 2023 [1] | Quasi-RCT | South Africa | 1489 | General population | App-based (HIVSmart!) | Participants used HIVSmart! to upload their self-test result and aid in test result interpretation. |
| Brady et al., 2016 [2] | Cross-sectional | United Kingdom | 3259 | General population | Web-based (PEBLFeedback.com) | Clients bought a blood-based HIVST online and from various outlets, then were able to provide feedback on site or via an independent website (PEBLFeedback.com). |
| Doan et al., 2021 [3] | Cross-sectional | United States | 271 | 18–30-year-old Black and Latino MSM | Web-based | Participants ordered an oral-based OraQuick test online (recruited via ads on social media/informational sites/dating sites) conducted the self-test, then took pictures of result and uploaded to online platform where two trained researchers analyzed results. |
| Kwan et al., 2023 [4] | Cross-sectional | China | 442 | 18+ year old MSM | Web-based | Participants were recruited through web-based channels and could then invite their peers to participate. Implementation cascade: 1) enrolment with questionnaire completion, 2) oral-based or blood-based self-test kit request, 3) test result upload, 4) web-based training, 5) peer referral. They could also ask for real-time support, including in-person, video call, and instant messaging support, at kit request. Participants requesting real-time support received a text message through instant messaging apps to schedule a time for the self-test. A support hotline was available to all study subjects. The referees could register on the same platform and go through the same steps. |
| O’Byrne et al., 2022 [5] | Cohort | Canada | 604 | General population | Web-based (GetaKit.ca) | Participants registered on GetaKit.ca and ordered a free blood-based bioLytical INSTI® HIVST to their home or designated pick-up location. Through the website, participants received a link to an online instructional video and were encouraged to report their results via the website. |
| Roche et al., 2024 [6] | Cross-sectional | Kenya | 1500 | 18+ year old general population | Web-based (CommCare) | Clients were given a Mylan blood-based HIVST kit and asked if they preferred performing the test on their own or with assistance on any aspect of the test except result interpretation. Once the test was complete, the research assistant photographed the test result using a Samsung Galaxy A6 tablet and the image was uploaded to the study’s secure electronic data collection platform (CommCare). |
| Wang et al., 2020 [7] | Cohort | China | 279 | 18+ year old MSM | Multi-modal (mobile phone applications, instant messaging chat rooms, blogs, and other websites) | Participants recruited via online advertising posting in apps, instant messaging chat rooms, blogs, and other websites. Also recruited via CDC clinics or by referral of study participants. Participants used an oral-based Aware™ HIVST and self-tests were conducted on-site. At follow-up, participants were again given pre-test counselling, and could choose to perform an oral HIVST or receive a blood HIV test or both. Participants again completed a questionnaire and received post-test counselling. |

Table S2: Study characteristics of secondary outcomes studies

| **Reference** | **Outcome(s)** | **Study Design** | **Country** | **Sample Size** | **Population** | **Digital Innovation** | **Intervention Description** |
| --- | --- | --- | --- | --- | --- | --- | --- |
| Bell et al., 2021 [8] | Preference Feasibility Impact | Cross-sectional | Australia | 794 | 18+ year olds general population - focused on MSM | Web-based (HIVST online ordering webpage with links to relevant HIV-related resources and follow-up telephone interview) | A study registration page was hosted on the established QPP website, which linked to an online order system for the HIVST kit. The webpage also included links to resources relating to HIV, testing, and living positive, along with referral and support services for people newly diagnosed with HIV. Once registered, participants were offered three pre-test information options, then the oral-based OraQuick HIVST kits were sent. Two weeks after mailing HIVSTs, post-test follow-up via telephone were conducted to check if kit was received, if test was completed, whether there were issues performing/reading the self-test, result of the test, and for participant to ask any questions. |
| Birdthistle et al., 2022 [9] | Acceptability Feasibility  Impact | Cross-sectional | South Africa | 3431 | 15–24-year-olds males and females | Multi-modal (MTV Shuga series on television, radio, and accompanying multimedia activities) | The participants completed an online survey, offered through Facebook, Instagram, and social media platforms of schools, universities, community groups, and clinics in Mthatha. The survey referred to their exposure to the MTV Shuga series campaign on HIV prevention including HIVST and PrEP. |
| Chan et al., 2021 [10] | Acceptability Feasibility Impact | Cohort | China | 350 | 18+ year old MSM | Web-based HIVST with online real-time counselling | Participants received the oral-based Aware™ HIVST kit, then made appointments through the HIVST-online administrators. Through video-chat, the administrators explained how to use the HIVST and sent them a demonstration video if needed. Pre-test counselling was provided, and participants performed the HIVST under online real-time supervision then showed the result to the administrator. |
| Doan et al., 2021 [3] | Feasibility Impact | Cross-sectional | United States | 271 | 18–30-year-old Black and Latino MSM | Web-based | Participants ordered an oral-based OraQuick test online (recruited via ads on social media/informational sites/dating sites) conducted the self-test, then took pictures of result and uploaded to online platform where two trained researchers analyzed results. |
| Fischer et al., 2021 [11] | Acceptability Feasibility Impact | Cross-sectional | South Africa | 751 | 18+ year old general population | Mobile app  (Ithaka) | Participants received their oral-based OraQuick HIVST from an HIV South-Africa distribution and research program. Peer educators then approached participants about participating in the study - if consent was received, peer educator helped the participant log into and register for the Ithaka app on the participant's phone, where they were able to report their self-test results. |
| Girault et al., 2021 [12] | Acceptability Preference Impact | Cross-sectional | Thailand | 2504 | 15+ year old MSM and transgender women | Web-based | Participants were able to choose unassisted HIVST, which they performed using the oral-based OraQuick self-test. Participants were given the HIVST along with a unique identifier code used to access secure pages on the Thai-language study website with a step-by-step video on the HIVST, an online questionnaire, and a place to report results. |
| Guadamuz et al., 2022 [13] | Feasibility  Impact | Cross-sectional | Thailand | 303 | 18+ year old MSM | Web-based (STOS) | Participants were sent the 4^th^ generation Alere Determine^TM^ blood-based HIVST and received online support from a counsellor while performing the self-test via online video conferencing |
| Howells et al., 2023 [14] | Preference  Impact | Cross-sectional | England | 14,923 | General population | Web-based | Participants were able to order a test through the programme and then offered a choice between self-testing and self-sampling. Those who chose self-test reported their HIVST result online. SMS reminders were also sent out to encourage users to return their kit or report their result. |
| Jin et al., 2021 [15] | Feasibility  Impact | RCT | China | 560 | MSM | Web-based | The control group assignment was standard HIV counselling, whereas the intervention group received online HIVST kit distribution. |
| Johnson et al., 2022 [16] | Feasibility Impact | Cohort | United States | 2022 | 18+ year old MSM living in New York state outside of NYC | Social medias | Participants were recruited via media campaign advertisements on popular social media/networks. Those who were eligible were asked for an email address to receive a coupon that they could redeem for a an oral-based OraQuick HIVST delivered to their home for free. Follow-up online surveys took place at four to eight weeks after completion of the eligibility survey. |
| Kaneko et al., 2022 [17] | Acceptability Impact | Cross-sectional | Japan | 224 | MSM | Digital vending machines | Self-administered paper-based questionnaires were completed by participants to determine the acceptability of using DVMs to distribute blood-based self-tests. Participants were divided into two groups based on whether they had ever undergone an HIV test. |
| Kaptchuk et al., 2024 [18] | Feasibility  Impact | Cross-sectional | India | 5324 | 18+ year old general population | Web-based (Safe Zindagi) | Through the website, participants could register and order a MORCHEK 3^rd^ generation oral mucosal transudate rapid HIVST kit. Virtual counsellors were also accessible through the website for support. Animated videos with voiceover were sent to participants via the counsellor through the website, which outline the HIVST steps. Once tested, participants could upload a picture of their HIVST result to the website and counsellors could stay in touch with testers via SMS/WhatsApp for pre- and post-test counselling. |
| Kwan et al., 2023 [4] | Preference Feasibility Impact | Cross-sectional | China | 442 | 18+ year old MSM | Web-based | Participants ordered an oral-based OraQuick test online (recruited via ads on social media/informational sites/dating sites) conducted the self-test, then took pictures of result and uploaded to online platform where two trained researchers analyzed results. |
| Larsson et al., 2023 [19] | Preference  Impact | RCT | Zimbabwe | 2181 | Youth aged 16-24 | Mobile app (ITHAKA) | The app was accessible through a web browser on smartphones or tablets, through which participants could submit their HIVST result. As well, the app included educational videos about HIV and HIV testing, four step-by-step videos on how to conduct the OraQuick OMT test, mapping of local HIV prevention care and services, chat/phone support, and journey tracking/reminders, and result interpretation. |
| Li et al., 2021 [20] | Impact | Cross-sectional | China | 1816 | 16+ year old MSM | Social medias (WeChat social media platform or Blued and other social medias) | Participants paid a 7$ deposit that would be reimbursed once they uploaded their test result and followed up with post-test counselling. Blood-based HIVST were sent to participants by mail. Participants received $2 once they uploaded their test results and alters were asked to enter IPs information so those who recruited alters received an extra $3. |
| Logie et al., 2023 [21] | Feasibility | RCT | Uganda | 152 (digital intervention arm) | Youth aged 16-24 | SMS | There were three arms, one of which had digital intervention (HIVST+). In this arm, participants received the OraQuick Rapid HIV-1/2 Antibody Test and were sent check-in messages through the web-based SMS platform. |
| Ly et al., 2023 [22] | Preference | Cross-sectional | Canada | 7712 | General population | Mobile app (I’m Ready, Test) | The mobile app provides access to free HIVST kits and continuative care. Pre-/post-test surveys were conducted to collect information of the participants’ perceived benefits of the app and barriers to HIVST such as healthcare access and COVID-19 restrictions. |
| MacGowan et al., 2024 [23] | Impact | RCT | United States | 2195 | 18+ year old Black, African American, Hispanic, and Latino MSM and transgender women | Mobile app (Know@Home) | The app was used to collect test data. Participants who had completed an OraQuick In-Home HIV test were presented with images and descriptions of the self-test results and asked to select the response that most aligned with their completed test. As well, participants could take and upload a picture of their self-test result to the app. |
| Maraj et al., 2023 [24] | Acceptability | Cross-sectional | Canada | 1269 | General population | Web-based (I’m Ready, Test) | Participants were able to book an appointment with peer navigators through the telehealth platform and receive counselling before, during, and after their HIVST. |
| Marley et al., 2021 [25] | Acceptability Impact | Cross-sectional | China | 692 | 16+ year old MSM | Web-based (wjx.cn) | Participants watched a short video on a smart-phone electronic readers (SER) prototype, were given a short introductory paragraph on SERs, and were asked questions on their willingness to use the prototype for HIVST. |
| McFall et al., 2023 [26] | Acceptability  Impact | Cross-sectional | India | 5014 | General population | Web-based | Virtual counsellors were available to participants for pre-/post-test counselling, assistance with using the HIVST kits (unspecified type), interpreting and uploading the test result to the website, and linked participants with continuation services. |
| Mshweshwe-Pakela et al., 2022 [27] | Feasibility Impact | Cross-sectional | South Africa | 2267 | 18+ year old general population | Tablet application | Six HIVST booths were set up in two clinics (three in each) with pictorial instructions to guide the self-testing process, the oral-based OraQuick HIVST, a tablet device with the app, and headphones for participants to listen to audio content on the app. The app guided the participants HIVST process including pre-test counselling, testing with a video demonstration how to use the self-test, and post-test counselling which included next steps after a negative or positive test. |
| Nguyen et al., 2024 [28] | Feasibility  Impact | Cross-sectional | Viet Nam | 17,589 | General population | Web-based | Participants could create an account on the website, on which they could fill out a form to order the OraQuick HIV Self-Test and complete a risk-assessment. The website provided details on how to perform the HIVST and report their results on the platform. |
| Ni et al., 2024 [29] | Feasibility  Impact | Quasi-RCT | China | 393 | 18+ year old MSM | Web-based (WeChat) | Sexual health influencers were index participants in the study, who connected participants to HIVST through secondary distribution. The WeChat platform was accessible to the index participants where they could see HIVST-related information, as well as order their self-test (unspecified type). All testers could submit their result by uploading an image of their result to the platform via a QR code that was in the HIVST package. |
| Ni et al., 2021 [30] | Impact | Cross-sectional | China | 1265 | MSM | Web-based | Participants could order up to five HIVST (unspecified type) through the online platform. Participants (indexes) were encouraged to distribute HIVST to members (alters) within their social networks. All were given a refund once test results were uploaded. |
| Ntinga et al., 2022 [31] | Acceptability Preference | Cross-sectional | South Africa | 120 | 18+ year old general population residing in Vulindlela or neighbouring community | Mobile app (Nolwazi_bot isiZulu - speaking conversational agent) | Participants were left alone in the testing room and provided the oral-based BioSure HIVST kit with instructions but were asked to only use the chatbot and instructions if the chatbot said to. Individuals could choose 1/4 personalities for their counsellor. Once prepared, the chatbot showed a video of how to use the test kit and interpret the results. Users then interpreted their result and reported it to the app. |
| O’Byrne et al., 2024 [32] | Feasibility  Impact | Cohort | Canada | 3627 | 16+ year old general population | Web-based (GetaKit.ca) | Participants could access and register on the website, where they also performed a risk assessment and were able to report their INSTI HIV self-test result. Those who provided a result were given information about retesting and PrEP, as well as given a referral for PrEP and/or confirmatory testing and linkage to care from registered nurses. |
| O’Byrne et al., 2023 [33] | Impact | Cross-sectional | Canada | 1551 | 16+ year old general population- focusing on MSM and ACB | Web-based (GetaKit.ca) | Participants were able to use the GetaKit website to order a free blood-based BioLytical INSTI® HIVST kit. Participants were asked to report their self-test results via the website. |
| O’Byrne et al., 2023 [34] | Impact | Cross-sectional | Canada | Over 5000 | General populations | Web-based (GetaKit.ca) | Participants used the web-based platform which used an algorithm to assess HIV specific risk factors and indications of HIV testing. As well, participants can order HIVST through the platform. |
| O’Byrne et al., 2022 [5] | Feasibility Impact | Cohort | Canada | 604 | General population | Web-based (GetaKit.ca) | Participants ordered an oral-based OraQuick test online (recruited via ads on social media/informational sites/dating sites) conducted the self-test, then took pictures of result and uploaded to online platform where two trained researchers analyzed results. |
| O’Byrne et al., 2021 [35] | Feasibility Impact | Cross-sectional | Canada | 399 | 18+ year old general population | Web-based (Getakit.ca) | Participants registered and ordered a blood-based BioLytical INSTI® HIVST through the GetaKit website. Participants were requested to, but not required to, upload their self-test results on GetaKit.ca |
| O’Byrne et al., 2021 [36] | Feasibility  Impact | Cross-sectional | Canada | 527 | 18+ year old general population | Web-based (Getakit.ca) | Participants used the GetaKit platform to order the self-tests, receive counselling, and receive linkage to care including confirmatory testing and treatment, or prevention (PrEP). |
| Oladele et al., 2023 [37] | Acceptability  Impact | Cross-sectional | Nigeria | 164 | Adolescents and young adults (ages 14-24) | Multi-modal (mobile app and SMS) | Participants were given a code, which they entered into their mobile phone. Once confirmed, a third-party web-based app is displayed on the user’s screen – all is backed onto an unstructured supplementary service data (USSD) platform. The USSD then requires the user to fill out key information, including what number of lines appears on their OraSure oral-based HIVST after testing, as well as provides post-test counselling via phone number for the participant. Once the test is completed, the participant received their test result via SMS. |
| Pai et al., 2021 [38] | Impact | Quasi-RCT | South Africa | 3095 | 18+ year old township populations | Mobile app (HIVSmart!) | Participants in the conventional arm were subjected to conventional HIV testing, and participants in the intervention arm conducted the oral-based OraQuick HIVST along with the support of the HIVSmart! application. Participants in the intervention arm were able to choose between a supervised or unsupervised option for self-testing. Participants were also able to upload their self-test result using the app, as well as receive linkage to care. |
| Phatsoane et al., 2023 [39] | Acceptability Feasibility Impact | Cross-sectional | South Africa | 9505 | 18+ year old general population | Multi-modal (mHealth system, SMS messaging) | At enrolment, participant details were entered into the mHealth system of Viamo Mobile, and participants were encouraged to conduct a short survey via recorded phone line or website to report their self-test (unspecified type) use and result. The system encouraged self-reporting through two SMS messages sent at three- and five-days post-registration. If not completed by day seven, an interactive voice response system called the participant to go through the survey and report their test result. |
| Pollard et al., 2022 [40] | Feasibility  Impact | Cross-sectional | India | 2610 | 19-45 year old men, women, and transgender women | Web-based platform | Participants were able to order the HIVST kits and contact counsellors via the platform. |
| Ramos et al., 2021 [41] | Acceptability | RCT | United States | 322 | Between 18–34-year-old sexual minority men of colour | Web-based (infographic) | Participants in the control group were given written HIVST instructions, whereas those in the intervention group were given a digital infographic pertaining to the instructions of oral-based HIVST. Participants completed the self-tests and data was collected using an online web-based survey in Qualtrics. |
| Rosadino et al., 2023 [42] | Acceptability Feasibility Impact | Quasi-RCT | Philippines | 1690 | 18–49-year-old MSM and transwomen residing in Metro Manila | Multi-modal (online channels of TheLoveYourself, Inc. and online messaging system) | Recruitment took place online and via online messaging systems to gather information from participants and deliver their blood-based SURE CHECK® HIVST kits. A virtual assistant system was available to participants. |
| Rourke et al., 2023 [43] | Impact | Cross-sectional | Canada | 5000 | 18+ year old general population | Mobile app (I’m Ready, Test) | Participants used the app to anonymously order free HIVST kits for home delivery or pick-up. |
| Shaikezhanov et al., 2021 [44] | Feasibility  Impact | Cross-sectional | Kazakhstan | 7315 | MSM and transgender people | Web-based (AmanBol) | The platform was used to order HIVST and receive by delivery, receive online support, and articles were published on the website to provide information on sexual and mental health. |
| Shrestha et al., 2024 [45] | Feasibility  Impact | Cohort | United States | 4859 | 18+ year old general population | Web-based (TakeMeHome.org) | Participants could use the online request platform to order their OraQuick In-Home HIV Test. |
| Shrestha et al., 2023 [46] | Acceptability  Feasibility  Impact | Quasi-RCT | Malaysia | 50 | 18+ year old MSM | Mobile app (JomPrEP) | Once the app was downloaded on their smartphone, participants were asked to create log-in credentials and were then directed to the home page of the app. Features of the app included the ability to order the OraSure HIVST, PrEP consultation, order monitoring, viewing their lab test result, making appointments, and messaging clinic or research staff. |
| Stafylis et al., 2022 [47] | Feasibility Impact | Cohort | United States | 254 | 18-30 years old MSM - Latinx or Black/African American | Multi-modal (social media, dating apps, and information search sites - leading to web-based platform for online test ordering) | Advertisements promoting free HIV self-testing were placed on social medias (Facebook, Instagram), dating apps (Grindr, Hornet), and information search sites (Google, Bing). Participants who clicked on the study advertisement and underwent eligibility criteria received a unique electronic code to order the oral-based OraQuick HIVST through Orasure.com. They were followed up 14 and 60 days after enrolment, and at follow-up, participants were asked about their HIVST use and result. |
| Thakker et al., 2022 [48] | Feasibility Impact | Cross-sectional | India | 1356 | General population | Web-based (www.safezindagi.net/selftesting) | Virtual outreach workers contacted clients on dating apps and social media platforms and provided counselling. As well, directed participants to HIVST (type unspecified) via the www.safezindagi platform that allows for home delivery or pick-up at a community site. HIVST could be assisted or unassisted with pre/post-counselling from virtual workers. |
| Tharao et al., 2022 [49] | Impact | Cross-sectional | Canada | 255 | 18+ ACB population | Mobile app (I’m Ready, Test) | Participants were able to use the platform to provide consent, conduct pre-/post-test surveys, order up to three HIV self-tests for delivery or pick-up, take the test, upload their result, and access a pee navigator for support before, during, and after the testing process. |
| Vasconcelos et al., 2022 [50] | Preference Impact | Cross-sectional | Brazil | 6477 | 18+ year old MSM | Web-based (A Hora e Agora-SP) | After participants had completed a web-based questionnaire, they were offered an oral-based OraQuick HIVST, free of charge. The project platform was then used to provide HIVST video instructions. |
| Vlasiuk et al., 2022 [51] | Feasibility  Impact | Cross-sectional | Ukraine | 21,035 | General population – focused on men | Multi-modal (web-based and digital VMs). | HIVST kits were distributed using social workers, two websites, and 10 vending machines. |
| Young et al., 2022 [52] | Feasibility | RCT | United States | 900 | 18+ year old Latinx and African American MSM living in LA | Social media (peer-led online support group) | After participants had completed a web-based questionnaire, they were offered an oral-based OraQuick HIVST, free of charge. The project platform was then used to provide HIVST video instructions. |
| Zhou et al., 2023 [53] | Feasibility  Impact | RCT | China | 300 (intervention group) | General population | Web-based | The intervention group was given a link to order two free quadruplex self-testing kits online. The HIVST tested for HIV-1 antibody, as well as antibodies of other diseases. Participants were asked to upload a picture of their test result within 48h using a Quick Response code. Reminder messages were sent to participants who had not uploaded their result within one week of testing. |
| Zhou et al., 2022 [54] | Feasibility Impact | RCT | China | 309 | 18+ year old MSM | Web-based  (HIVST online ordering system developed by Xutong) | An online HIVST ordering system was used, which was hosted and managed using WeChat. The study used the blood-based SD BIOLINE HIV/syphilis self-test. The control group was refunded for the HIVST, whereas the SD-M group could receive $3 per self-test, and the SD-M-PR group also could receive $3 per self-test and could refer up to 10 alters to receive a maximum of $30. |
| Zhu et al., 2024 [55] | Feasibility  Impact | Quasi-RCT | China | 28 | 18+ year old MSM | Mobile app (WeTest+) | Participants were added to the WeTest+ platform and asked to follow the content for three weeks. They were asked to read messages from the WeTest+ platform library, use the interactive content (including quizzes, information about local HIV services, and access to counsellors). Participants were asked to perform their finger-prick or oral-based (manufactured by Beijing Kewei Clinical Diagnostics Reagent Inc.) self-test and to upload a photo of their test result using a private QR code. |

Table S3: Risk of bias assessment of accuracy studies using the QUADAS 2 Tool – low risk = ☺, high risk = ☹, unclear = ?

| Study | Patient Selection | Index Test | Reference Standard | Flow and Timing |
| --- | --- | --- | --- | --- |
| Beecroft et al., 2023 [1] | ☺ | ☺ | ☺ | ? |
| Brady et al., 2016 [2] | ? | ☺ | ? | ? |
| Doan et al., 2021 [3] | ☺ | ☺ | ? | ? |
| Kwan et al., 2023 [4] | ☺ | ☺ | ? | ? |
| O’Byrne et al., 2022 [5] | ☺ | ☺ | ? | ? |
| Roche et al., 2024 [6] | ☺ | ☺ | ? | ? |
| Wang et al., 2020 [7] | ☺ | ☺ | ☺ | ☺ |

Table S4: Risk of bias assessment of RCTs using the Cochrane Risk of Bias Tool 2 - low risk = ☺, some risk = 😐, high risk = ☹

| Study | Randomization process | Deviations from intended interventions | Missing outcome data | Measurement of outcome | Selection of reported result |
| --- | --- | --- | --- | --- | --- |
| Jin et al., 2021 [15] | ☺ | ☺ | 😐 | ☺ | ☺ |
| Larsson et al., 2023 [19] | ☺ | ☺ | ☺ | ☹ | ☺ |
| Logie et al., 2023 [21] | ☹ | ☺ | ☺ | 😐 | ☺ |
| MacGowan et al., 2024 [23] | ☹ | ☺ | ☺ | ☺ | ☺ |
| Ni et al., 2024 [29] | 😐 | ☺ | ☺ | ☺ | ☹ |
| Pai et al., 2021 [38] | 😐 | ☺ | ☺ | ☺ | ☺ |
| Ramos et al., 2021 [41] | ☺ | ☺ | ☺ | ☺ | ☺ |
| Rosadino et al., 2023 [42] | ☹ | ☺ | ☺ | ☺ | ☺ |
| Shrestha et al., 2023 [46] | ☹ | ☺ | ☺ | 😐 | ☺ |
| Young et al., 2022 [52] | ☺ | ☺ | ☺ | ☺ | ☺ |
| Zhou et al., 2023 [53] | 😐 | ☺ | ☺ | 😐 | ☹ |
| Zhou et al., 2022 [54] | ☺ | ☺ | ☺ | ☺ | ☺ |
| Zhu et al., 2024 [55] | ☹ | ☺ | ☺ | 😐 | ☺ |

Table S5: Risk of bias assessment of cross-sectional studies using the Newcastle-Ottawa Scale

| Study | Selection  (maximum 5) | Comparability  (maximum 2) | Outcome  (maximum 3) | Overall  Quality |
| --- | --- | --- | --- | --- |
| Bell et al., 2021 [8] | *** | ** | ** | Good |
| Birdthistle et al., 2022 [9] | **** | ** | * | Good |
| Doan et al., 2021 [3] | * | * | ** | Fair |
| Fischer et al., 2021 [11] | *** | ** | ** | Good |
| Girault et al., 2021 [12] | *** | ** | ** | Good |
| Guadamuz et al., 2022 [13] | * | N/A | * | Poor |
| Howells et al., 2023 [14] | ** | N/A | * | Poor |
| Kaneko et al., 2022 [17] | ** | ** | ** | Fair |
| Kaptchuk et al., 2024 [18] | **** | N/A | ** | Poor |
| Kwan et al., 2023 [4] | *** | ** | ** | Good |
| Li et al., 2021 [20] | ** | ** | *** | Good |
| Ly et al., 2023 [22] | ** | * | ** | Fair |
| Maraj et al., 2023 [24] | ** | ** | * | Fair |
| Marley et al., 2021 [25] | ** | ** | ** | Fair |
| McFall et al., 2023 [26] | ** | N/A | * | Poor |
| Mshweshwe-Pakela et al., 2022 [27] | * | * | *** | Fair |
| Nguyen et al., 2024 [28] | *** | N/A | *** | Poor |
| Ni et al., 2021 [30] | ** | N/A | ** | Poor |
| Ntinga et al., 2022 [31] | * | ** | ** | Fair |
| O’Byrne et al., 2023 [33] | *** | * | ** | Fair |
| O’Byrne et al., 2023 [34] | ** | N/A | * | Poor |
| O’Byrne et al., 2021 [35] | *** | ** | ** | Good |
| O’Byrne et al., 2021 [36] | ** | * | * | Fair |
| Oladele et al., 2023 [37] | 0 | N/A | *** | Poor |
| Phatsoane et al., 2023 [39] | ** | ** | ** | Fair |
| Pollard et al., 2022 [40] | ** | ** | ** | Fair |
| Rourke et al., 2023 [43] | ** | ** | ** | Fair |
| Shaikezhanov et al., 2021 [44] | ** | * | * | Fair |
| Thakker et al., 2022 [48] | * | ** | * | Fair |
| Tharao et al., 2022 [49] | ** | ** | * | Fair |
| Vasconcelos et al., 2022 [50] | ** | ** | ** | Fair |
| Vlasiuk et al., 2022 [51] | ** | ** | * | Fair |

Table S6: Risk of bias assessment of cohort studies using the Newcastle-Ottawa Scale

| Study | Selection  (maximum 4) | Comparability  (maximum 2) | Outcome  (maximum 3) | Overall  Quality |
| --- | --- | --- | --- | --- |
| Chan et al., 2021 [10] | **** | ** | *** | Good |
| Johnson et al., 2022 [16] | *** | ** | * | Fair |
| O’Byrne et al., 2024 [32] | *** | * | * | Poor |
| O’Byrne et al., 2022 [5] | *** | ** | * | Fair |
| Shrestha et al., 2024 [45] | *** | N/A | * | Poor |
| Stafylis et al., 2022 [47] | *** | N/A | * | Poor |

Table S7: Key findings of accuracy studies

| **Reference** | **Key Findings** |
| --- | --- |
| Beecroft et al., 2023 [1] | **Accuracy (all metrics):** results from HIVST were compared to a reference standard of two rapid tests and one HIV RNA and the following were measured: Specificity of 99.9% (95% CI: 99.8%-100.0%), sensitivity of 95.5% (95% CI: 94.5%-96.6%), PPV of 99.2% (95% CI: 98.8%-99.7%), and NPV of 99.6% (95% CI: 99.2%-99.9%). |
| Brady et al., 2016 [2] | **Accuracy (invalids):** Reported rate of <0.2% for invalid tests. **Accuracy (specificity)**: the rate of false positives was 3 but was expected to be 25. |
| Doan et al., 2021 [3] | **Accuracy (agreement):** Proportion of result agreement among reviewers was 113/113 (100%, k=1.0). proportion of result agreement between reviewers and participants was 110/113 (97.3%, k=0.85 95% CI 0.67-1.0). **Accuracy (invalids):** 2/113 (1.8%) of concordant results were invalid. |
| Kwan et al., 2023 [4] | **Accuracy (invalids):** 17/394 returned kits (4.3%) were invalid. **Accuracy (sensitivity):** Of the positive results, 2/4 were confirmed to be true positives (one was already on ART). **Accuracy (not specified):** Accuracy of the participants result interpretation was 99.1% (95%CI 97.4%-99.8%). |
| O’Byrne et al., 2022 [5] | **Accuracy (invalids):** 81/604 participants reported 89 invalid results. 5/81 participants did not reorder a self-test. 6 participants reported 2 invalid results, resulting in 12/89 of all reported invalids (13%). Reported rate for invalid tests for all tests ORDERED was an average of 12% (0%-22%). Reported rate for invalid tests for all tests REPORTED was an average of 22% (0%-38%). Excluding the 6 participants who each reported 2 invalid tests (and 18 orders they placed), invalid tests were 9% of all ordered tests and 12% of all reported results. Invalid rate dropped after the peak in late May 2021 (when they started sending detailed instructions about completing the ST). Invalids continued to be over 10%. |
| Roche et al., 2024 [6] | **Accuracy (all metrics):** 854 images were completed by participants and sufficient to be interpreted by the AI algorithm. Specificity was high, 98.8% (95% CI 98.0%-99.5%), as 798/808 of the negative tests were accurately identified. Sensitivity was 100.0% as all 44 positive tests were correctly classified as positive. PPV was lower at 81.5% (95% CI 71.1%-91.8%), whereas NPV was also high at 100.0%. |
| Wang et al., 2020 [7] | **Accuracy (all metrics):** Specificity was high, 96.8% (121/125), among MSM recruited via the internet. Sensitivity was lower, 92.9% (13/14), PPV was 76.5% (13/17), and NPV was 99.2% (121/122). |

Table S8: Key findings of secondary outcomes studies

| **Reference** | **Key Findings** |
| --- | --- |
| Bell et al., 2021 [8] | **Preference:** Participants reported that the reasons they chose to test for HIV via the online HIVST project were due to convenience (79%; 726), not wanting to wait for results (44%; 402), not wanting to talk about sex with anyone (33%; 298), not having time to go elsewhere for a test (29%; 268), and fear of stigma (22%; 205). Lack of local HIV testing services was reported for 7.2% (66) of orders. Of the 190 first order participants who responded to the survey question “Where would you have tested if HIVST was not available?”, 21% (40) reported they would not have tested elsewhere. **Feasibility (uptake):** During the study period, 95 (14%) participants ordered two or more HIVST kits (range 2–7, median 2, interquartile range (IQR) 2–3 HIVST kits). **Feasibility (response rate):** Post-test peer worker contact with participants was achieved for 52% (485) of HIVST orders. Despite three attempts to contact participants, 48% (440) were unable to be contacted. **Impact (new infections and linkage to care):** One participant reported a reactive result during the study period; with the support of the resources provided in the HIVST kit, the participant successfully self-navigated their way to confirmatory HIV testing and linkage with an HIV healthcare provider prior to the two-week follow-up call. During the follow-up telephone call, the PTF was able to link the participant with the QPP Peer Navigation Program. **Impact (first-time testers):** No previous HIV test was reported by 45% (353) of first order participants. Almost one-third (31%; 123) of the men who only had sex with men reported having never tested for HIV, compared with 59% (56) of men who had sex with men and women (MSMW) (24.356, p < 0.001). The odds of ever having had an HIV test were decreased by 30% for MSMW (OR 0.3, 95% CI 0.2–0.5) compared to MSM. |
| Birdthistle et al., 2022 [9] | **Acceptability (willingness):** Among those who had never self-tested, 83% were interested in using an HIVST, and interest in giving a ST kit to a partner was high (not specified). **Feasibility (visits to web-based provider):** Overall 43% had engaged with the campaign. **Impact (first-time testers):** Proportion of those who had used an HIVST at any point in their life (29% vs 10%; aOR=2.49 (1.95 to 3.19)) or sometime within the past year (21% vs 7%; aOR=2.61 (1.97 to 3.47)) was higher among those exposed vs those unexposed to campaign. Out of the 2694 participants who responded, 211/2094 of the unexposed and 186/645 of the exposed HAD tested for HIV using an HIV self-screening kit. |
| Chan et al., 2021 [10] | **Acceptability (ease of use):** Out of 125 people who completed the process evaluation, 72.0%-97.6% believed that the online real-time counselling was helpful in different aspects such as understanding their current risk, testing results, concept of window period, and reducing their fear toward HIV testing and high-risk behaviours. **Feasibility (uptake):** 40.4% (92/228) of new-users and 63.1% (77/122) of ever-users received the HIVST-online during the project period. **Impact (new infections and linkage to care):** 4 HIVST-online users were screened to be HIV positive, all of who received confirmatory tested (facilitated by administrators) and were confirmed to be HIV positive. **Impact (not specified and first-time testers):** 16.2% of the 228 new-users of HIVST-online had NOT been tested for HIV in the past 3 years. 19.7% of ever-users of HIVST-online had NOT been tested for HIV in the past 3 years. 17.4% of all users had not tested for HIV in the past 3 years. |
| Doan et al., 2021 [3] | **Feasibility (uptake):** 191/271 (70.5%) of participants ordered an HIVST, and 159/191 (83%) of those used it. **Impact (new infections):** 7/113 (6.2%) of concordant results were positive. **Impact (result return):** 113/159 (71%) submitted readable test result images. |
| Fischer et al., 2021 [11] | **Acceptability (ease of use):** Likert scores of 3.8 (SD-1.6) for "made it easy to upload results" and 4.2 (SD=0.9) for "easy to find a clinic". Reasons participants stopped using the app included used the app to completion, and unable to upload their HIVST results (2 out of 41 people said this). **Feasibility (visits to web-based provider):** 531/751 (70.7%) of participants logged on to the app. **Impact (new infections):** 14/168 (8.3%) of those who self-reported their results were HIV positive. **Impact (result return):** 168/751 (22.4%) self-reported their results. |
| Girault et al., 2021 [12] | **Acceptability (willingness):** 2472/2504 (98.72%) of all participants said they were interested in HIVST in the future. **Preference:** 2486/2504 (99.3%) selected HIVST vs referral to HIV testing services. **Impact (new infections):** Only accounting for participants that opted for assisted or unassisted HIVST, 96/1405 (6.83%) MSM and 72/1070 (6.73%) transwomen tested positive. Among the referral group, 1/11 (9.1%) MSM and 1/7 (14.3%) of transwomen tested positive. All except one who tested positive were confirmed to be positive. **Impact (first-time testers):** 491/1422 (34.5%) of MSM and 414/1082(38.3%) of transwomen had never been tested for HIV. **Impact (linkage to care):** Among all who needed confirmatory testing, 108/179 (60.3%) were referred and accessed the HIV testing services, including 5 participants who had invalid results. Of those who tested positive with confirmatory testing, 91/104 (87.5%) were linked to treatment services. |
| Guadamuz et al., 2022 [13] | **Feasibility (uptake):** 267/303 (88.1%) of participants successfully conducted STOS at baseline. At the three-month follow-up, 153/175 (87.4%) of those who were sent test kits again successfully conducted STOS. **Impact (new infections):** At baseline, 69/267 (25.8%) of those who tested received a positive result. At the three-month follow-up, 153/153 (100%) of those who tested received a negative result. **Impact (linkage to care):** All participants (100%) who tested positive were immediately linked to HIV care, treatment, and support. |
| Howells et al., 2023 [14] | **Preference:** 68.0% (approximately 10,147/14,932) of participants chose a self-test over the self-sampling option. **Impact (result return):** 52% of the self-testers (approximately 5280/10,153) reported their self-test result. |
| Jin et al., 2021 [15] | **Feasibility (uptake):** 317/560 (56.6%) participants used the HIVST. 1556 HIVST were delivered by mail. **Impact (result return):** 1407/1556 (90.4%) HIVST kits reported results. **Impact (new infections):** 12/317 (3.8%) of those who used an HIVST received a positive result. **Impact (referrals):** 182 male partners conducted the HIVST by secondary distribution of study participants. |
| Johnson et al., 2022 [16] | **Feasibility (uptake):** 922/1114 participants who redeemed the coupon and completed the follow-up survey used the HIVST kit to test themselves. **Impact (new infections and linkage to care):** 7/922 (0.8%) of those who used the HIVST for themselves tested positive, 6/7 reported they had a confirmatory test, and 5/6 self-reported they were confirmed as HIV-positive and were linked to medical care (one was waiting for the confirmatory test results at the time of the follow-up survey). **Impact (first-time testers):** 976/3197 (30.5%) of eligible participants had never been tested for HIV. |
| Kaneko et al., 2022 [17] | **Acceptability (willingness):** Amongst MSM who had never been tested (N=37), 72.2% showed willingness to purchase tests from DVMs - even at the cost of 1000 Japanese yen. 10/37 (29.7%) knew about HIVST/postal DBS and 26 (70.3%) did not. 12 (33.3%) said they would "very much" use HIVST if it were free, 16 (44.4%) responded "pretty much", 8 (22.2%) responded "not so much", and 0 said they didn't want to. At a cost of 1000 Japanese yen, 3 (8.3%) said they would "very much" use HIVST, 19 (52.8%) responded "pretty much", 13 (36.1%) responded "not so much", and 1 (2.8%) didn't want to. Of those who had been tested before, 117/187 (63.7%) knew about HIVST and 65 (34.8%) did not. 89 (49.2%) said they would "very much" use HIVST if it were free, 51 (28.2%) responded "pretty much", 22 (12.2%) responded "not so much", and 19 (0.5%) said they didn't want to. At a cost of 1000 Japanese yen, 47 (26.1%) said they would "very much" use HIVST, 67 (37.2%) responded "pretty much", 39 (21.7%) responded "not so much", and 27 (15%) said they didn't want to. **Impact (first-time testers):** 37/224 participants had never been tested for HIV before. |
| Kaptchuk et al., 2024 [18] | **Feasibility (uptake):** 5324 unique participants registered and ordered an HIVST kit. The total number of kits ordered was 5840 since 7.0% of participants had reported orders. **Impact (new infections):** 75% (138/184) of those with a positive tests result who sought confirmatory care were confirmed to be positive. **Impact (first-time testers):** 53% (1897/3597) of those who responded about testing history had never tested before. **Impact (result return):** 93% (4607/4937) of all the kits received by participants were completed and had their result reported. Similarly, 94% (4282/4553) of participants who received their kits completed the test and uploaded their result. **Impact (linkage to care):** 72% (184/254) of participants who tested positive on the self-test completed confirmatory testing. 85% (117/138) of those who were confirmed positive cases were successfully linked to ART. |
| Kwan et al., 2023 [4] | **Preference:** At test kit request, most (338/434, 77.9%) did not opt for any support, while 18.9% (82/434), 1.8% (8/434), and 1.4% (6/434) requested instant messaging, video calls, and in-person support, respectively. Of those who accepted oral fluid tests only, they preferred getting tested for HIV in community-based organizations (OR 3.11, 95% CI 1.94-4.99, P<.001) to performing self-tests (OR 0.45, 95% CI 0.27-0.75, P=.002). The preferred modes of self-test support were instant messaging apps (77/155, 49.7%), in-person (74/155, 47.7%), and voice call (64/155, 41.3%), while video calls and chatbots were preferred by 7.7% (12/155) and 8.4% (13/155), respectively. **Feasibility (uptake):** Almost all (434/442, 98%) MSM who completed the questionnaire requested a self-test. **Feasibility (response rate):** More than half (216/354, 61%) of the eligible participants initiated the referral process by attempting the web-based training with a passing rate of 93% (200/216). Of the 200 participants who passed the web-based training, 111 (55.5%) eventually made at least one referral. **Impact (new infections):** Of 394 kits returned, 333 (94.1%) were negative, 4 (1.1%) were positive. **Impact (first-time testers):** 21.4% of participants had never been tested for HIV. Impact (return rate): Of those who requested a self-test, 82% (354/434) had uploaded their test results. |
| Larsson et al., 2023 [19] | **Preference:** 5.9% (128/2181) of participants chose self-testing versus HIV testing performed by the provider. 14.8% (19/128) of participants who self-tested chose to do so at home, meaning 85.2% (109/128) chose to do the test on-site. **Impact (result return):** 47.4% (9/19) of those who conducted the HIVST at home completed the test and reported their result. Comparatively, 99.1% (108/109) of the self-testers who did so on-site completed the app journey and reported their result. |
| Li et al., 2021 [20] | **Impact (new infections):** 51/1816 (2.81%) of participants had a positive HIVST result. **Impact (first-time testers):** 111/394 (28.2%%) of alters and 329/1422 (23.1%) of index participants had never been tested for HIV before. **Impact (result return):** 1816/2263 (80.25%) uploaded their HIVST result - 1422 (88.3%) index participants and 394 (21.7%) alters. **Impact (linkage to care):** Of those who sought HIV care (41/51), 35/41 received an HIV-positive confirmatory result and 34/35 of them started ART. |
| Logie et al., 2023 [21] | **Feasibility (uptake):** 30.9% (47/152) of HIVST+ participants conducted an HIVST at the baseline. Comparatively, 94.2% (113/120) and 94.2% (97/103) of these participants conducted the self-test at the 8 and 12 month follow ups, respectively. **Feasibility (response rate):** 60.2% (62/103) of participants who finished the study had responded to the two-way messaging platform. |
| Ly et al., 2023 [22] | **Preference:** Participants in rural areas reported a greater benefit of using the app (p<0.05). |
| MacGowan et al., 2024 [23] | **Impact (first-time testers):** 23.2% (509/2195) of participants had never been tested for HIV before. **Impact (result return):** 83.0% (1816/2195) of participants reported their HIVST result, in either the initial or 4-month survey. 31.0% (550/1816) of these participants reported their self-test result in the post-intervention survey. **Impact (referrals):** 78% (479/618) of participants who reported on distributing the HIVSTs had reported that they gave a self-test to a social network associate. In total, 491 HIVST were distributed to social network associates. |
| Maraj et al., 2023 [24] | **Acceptability (willingness):** over 80% of all participants reported high levels of satisfaction with the platform, saying that the knowledge of the peer navigators was the primary reason for their satisfaction, and they would use it again and recommend it to others. |
| Marley et al., 2021 [25] | **Acceptability (willingness):** 493/692 (71.2%) participants were willing to use a SER, 115/692 (16.6%) were unwilling, and 84/692 (12.1%) were unsure of their willingness. 483/493 (98%) of willing participants agreed that having an SER would increase their HIVST frequency. Reasons for willingness included obtaining accurate self-test results, ease of use, and short wait time of 15-20 minutes for results. Obstacles included cost of the reader and fear of test results leaking to others. Reasons for unwillingness included never having heard of the reader, and purchase cost. Some would consider using SERs due to its ease of use and less wait time of 15-20 minutes for results. **Impact (first-time testers):** 194/692 used the HIVST for their first HIV test ever. 80/692 (11.6%) of participants had "never" tested for HIV within the past year. 156/692 (22.5%) of participants had never self-tested for HIV, and 80/692 (11.6%) had never even heard of self-testing. |
| McFall et al., 2023 [26] | **Acceptability (ease of use):** 87.0% (approximately 265/305) survey respondents said the website was easy to use for ordering the HIVST kit. **Impact (new infections):** 5.0% (approximately 15/305) people who completed the follow-up survey stated they had a verified positive HIVST result. **Impact (result return):** 82.0% (approximately 4111/5014) participants uploaded their self-test result. |
| Mshweshwe-Pakela et al., 2022 [27] | **Feasibility (uptake):** The programme increased overall facility HIV tests by 25% (14.5% clients testing before compared to 19.9% testing during) while maintaining an HIV testing yield of 11%. **Impact (new infections):** 264/2267 (11.6%) were positive on the HIVST. 241/264 (91.3%) of those who tested positive received a confirmatory test and 230/241 (95.4%) were confirmed as positive. HIVST positivity yield was 12% (similar to traditional testing). The platform almost doubled the number of youths that were diagnosed with HIV (from 240 to 453). **Impact (linkage to care):** 150/230 (65%) initiated ART at the same clinic within 14 days, and 184/230 (80%) initiated ART within 9 months. |
| Nguyen et al., 2024 [28] | **Feasibility (uptake):** 11,332 unique people ordered 13,334 HIVST kits. **Impact (new infections):** 96.8% (522/539) of tests sent for confirmatory testing were confirmed to be positive. **Impact (first-time testers):** 44.9% (5069/11,332) of participants had never tested. 73.9% (9855/13,334) of the tests conducted were done by first-time testers. **Impact (result return):** 67.0% (8940/13,334) of the kits had a result reported. **Impact (linkage to care):** 539 tests were sent for confirmatory testing. As well, 97.5% (509/522) of confirmed HIV-cases initiated ART. Almost 20.0% of all participants who reported a negative test result had initiated PrEP. Overall, 23.4% (2095/8940) of completed tests with reported results led to ART or PrEP initiation. |
| Ni et al., 2024 [29] | **Feasibility (uptake):** 58.3% (229/393) of index participants ordered HIVST kits for secondary distribution, 224 of which were ordered through the platform in the first round. In this round, 573 kits were ordered, 62 of which were through peer referral links. In the second round, 85 index participants ordered 322 HIVST kits through the platform, 63 of which were through peer referral links. **Impact (new infections):** 1.9% (14/750) of returned results were confirmed as positive cases. **Impact (first-time testers):** 8.3% (19/229) of index participants had never tested for HIV. **Impact (result return):** 85.5% (488/573) of the tests had a result reported in the first round. 81.4% (262/322) of tests from the second round had a reported result. |
| Ni et al., 2021 [30] | **Impact (result return):** 1935/1984 (97.5%) results returned, of which 648/1935 were from 625 alters. |
| Ntinga et al., 2022 [31] | **Acceptability (ease of use):** 9/120 (7.5%) said the chatbot was easy to use. **Preference:** 95/120 (79.2%) said their HIV testing experience was much better with a chatbot than with a human counsellor, 14/120 (11.7%) said it was about the same, 7/120 (5.8%) said the experience was slightly better, and 2/120 (1.7%) felt the experience was much worse with the chatbot than that with a human counsellor. 93/120 (77.5%) said they felt as if they were talking to a real person, 15/120 (12.5%) said it did not feel as if they were chatting with a real person, and 12/120 (10%) did not respond to the question. Advantages for the chatbot included providing a safe space (no rush or judgement), offering HIV testing that is confidential, functionality, efficiency (do not have to wait at the clinic all day for results) (29/120 (24.2%) of all participants did not say any advantages when asked. Disadvantages included lack of empathy, if HIV-positive they would have the chance to hurt ("kill") themselves since they wouldn't receive the same care as a human counsellor, conversation was unidirectional, and it was easy to make a mistake (99/120 (82.5%) of all participants did not provide any disadvantages when asked. On a scale of 1-10 for preference of the chatbot, from the participants who responded (108/120), the average score was 9.32 (SD=1) ranging from 6-10. |
| O’Byrne et al., 2024 [32] | **Feasibility (uptake):** 5235 tests were ordered by 3627 unique participants. **Impact (new infections):** 0.4% (16/3627) of participants reported positive results. **Impact (first-time testers):** 38.9% (1334/3431) of participants were first-time testers. **Impact (linkage to care):** 100.0% (16/16) of those who tested positive were linked to confirmatory testing. |
| O’Byrne et al., 2023 [33] | **Impact (new infections):** There were five positive HIV self‐test results reported, split evenly among ACB and White participants. **Impact (first-time testers):** More White (79%) than ACB participants (70%) reported prior HIV testing, whether as serology, point‐of‐care testing, or self‐testing (X² = 8.97, p = 0.002). 21% of White participants, and 30% of ACB participants were first-time testers. **Impact (result return):** Among the 62% (n = 962/1551) of participants who reported their HIV self‐test results, more White (63%) than ACB (52%) participants reported their results (X² = 12.28, p < 0.001). |
| O’Byrne et al., 2023 [34] | **Impact (new infections/linkage to care):** 18 participants were found to be HIV positive, and all were linked to HIV treatment/care. **Impact (first-time testers):** 26% of participants reported never having conducted an HIV test in their lives. **Impact (result return):** 59% of participants reported their results. |
| O’Byrne et al., 2022 [5] | **Feasibility (uptake):** 604 participants ordered 701 HIVST. **Impact (first-time testers):** 25% of participants reported no previous HIV testing, and 4% were unsure if they had. |
| O’Byrne et al., 2021 [35] | **Feasibility (uptake):** 405/600 eligible participants (67.5%) ordered an HIVST, but 6 selected "prefer not to report" so were excluded from analysis. **Impact (new infections):** 1/399 (0.24%) person tested positive. **Impact (first-time testers):** 95/399 (23.9%) reported no primer testing and 13/499 (3.3%) were uncertain if they had ever previously been tested for HIV. **Impact (result return):** 228/399 (57.1%) of participants reported their HIVST results back through GetaKit.ca. |
| O’Byrne et al., 2021 [36] | **Feasibility (uptake):** 259/527 (49.1%) of participants ordered an HIVST kit. **Impact (new infections):** 0/182 (0.0%) of returned results were positive. **Impact (first-time testers):** 58/259 (26.3%) of those who ordered a test had never undergone HIV testing before. **Impact (result return):** 182/259 (70.3%) of those who tested reported their result. **Impact (linkage to care):** 159/259 (61.4%) of participants were referred for PrEP use. |
| Oladele et al., 2023 [37] | **Acceptability (ease of use):** 100.0% (164/164) of participants were able to conduct the self-test, interpret their results, and use the linkage to care platform. **Impact (new infections):** 0 new cases were diagnosed by confirmatory testing, although 7.9% (13/164) of participants had a positive result on the HIVST. **Impact (linkage to care):** 100.0% (13/13) of those who tested positive were linked to confirmatory testing. |
| Pai et al., 2021 [38] | **Impact (new infections):** 106/1560 (6.8%) of conventional arm and 136/1535 (8.9%) of the intervention arm (7.6% unsupervised and 10.9% supervised) tested positive for HIV. **Impact (linkage to care):** Almost all participants were linked to care (99.7% in unsupervised, 99.8% in supervised, and 98.5% conventional testing). ART for HIV-positive participants was initiated by 98.1% for intervention arm (95.7% supervised and 99.3% unsupervised) and 98.5% for conventional arm. |
| Phatsoane et al., 2023 [39] | **Acceptability** (**ease of use**)**:** 1592/2467 (64.5%) reported the HIVST was very easy or easy to use. **Feasibility** (**response rate**)**:** In total, 2,467/9505 (26.0%) participants answered any survey question. 237 (2.5%) participants of those who had not called before day three) participants called and completed the survey after receiving the SMS reminder message on day three, and before receiving the second reminder on day five. 123 (1.3%) of those who had not called before day five called after receiving a second reminder on day five. The remaining 8,109 (85.3% of total) of participants received a phone call seven days after enrolment by the same recorded phone survey as accessed by those calling into the system. Of these, 1,777 (20.7% of those called) answered the first question of the survey. **Impact** (**new infections**)**:** Out of 314 respondents reporting an HIV positive test, 130 (41.4%) reported that this was the first positive HIVST that they had taken. **Impact (result return):** 1,933/2467 (78.4%) of those (1933/9505, 20.3% of total) were willing to self-report their HIV status. 1,321/8109 (15.4% of those called) self-reported HIV status. **Impact** (**linkage to care**)**:** Of the 314 respondents reporting a HIV positive test, 204 (64.9%) reported that they had either linked to care or intended to link to care. |
| Pollard et al., 2022 [40] | **Feasibility (uptake):** 2610 ordered an HIVST and 2185 received the test kit. **Impact (new infections):** 93/2018 (4.6%) received a positive result. **Impact (result return):** 2018/2185 (92.4%) of participants reported their HIVST result. **Impact (linkage to care):** 31/93 (33.3%) of those who tested positive were linked to confirmatory testing, 25/31 (80.6%) were confirmed to be HIV positive, and 25/25 (100%) started ART. |
| Ramos et al., 2021 [41] | **Acceptability (ease of use):** 71.6% of participants agreed that the infographic was useful. 73.5% of participants agreed that the infographic was easy to use. 69.2% of participants agreed that the infographic was easy to learn. 71.6% of participants were satisfied with the infographic. Mean of "somewhat" agreeable that the infographic was useful (M=5.46, SD=1.40), easy to use (M=5.51, SD=1.23), easy to learn (M=5.41, SD 1.37) and satisfied with the infographic (M=5.34, SD=1.33). |
| Rosadino et al., 2023 [42] | **Acceptability (willingness):** out of 4205 respondents, 4163 (99.0%) were interested in getting an HIVST. **Feasibility (uptake):** 4009/4205 (95.3%) underwent pre-qualification process, and only 2543 (60.5%) were eligible, of which only 2232 (53.1%) were unique respondents. Only 1690 participants successfully received their HIVST kit. **Impact (new infections):** 93/953 participants tested positive on the HIVST (9.8%). **Impact (first-time testers):** 454/1690 (26.9%) who received the kit were first time testers. **Impact (result return):** 953/1690 (56.4%) reported their results. **Impact (linkage to care):** 56/93 (60.2%) were linked to further testing. |
| Rourke et al., 2023 [43] | **Impact (first-time testers):** 53% of those in the 18-24 age group were first-time testers, compared to 20%-30% of those in the higher age groups (OR=3.30, 95% CI=2.88,3.78). |
| Shaikezhanov et al., 2021 [44] | **Feasibility (uptake):** 7315 HIVST were delivered. **Feasibility (response rate):** 5048/7315 (69.0%) of feedback responses were received. **Feasibility (visits to web-based provider):** The website was viewed 266,000 times. **Impact (new infections):** 165 positive results were reported. **Impact (linkage to care):** 93/165 (56.4%) people were linked to care. |
| Shrestha et al., 2024 [45] | **Feasibility (uptake):** 5323 kits were ordered by 4859 participants. **Impact (new infections):** 0.6% (31/4859) of people reported a positive result from the HIVST. |
| Shrestha et al., 2023 [46] | **Acceptability (ease of use):** 98.0% (49/50) of participants reported that using the app to order an HIVST kit was easy. **Feasibility (uptake):** 84.0% (42/50) of participants ordered an HIVST kit through the app. **Impact (first-time testers):** 2.0% (1/50) participants were first-time testers for HIV. **Impact (linkage to care):** 92.0% (46/50) of participants used the app to begin taking PrEP. |
| Stafylis et al., 2022 [47] | **Feasibility (uptake):** 177 of the 254 participants ordered test kits during the study period. Overall, those recruited through dating apps had the highest order rate (1.24 kits/day), followed by social media platforms (0.24 kits/day) and information search platforms (0.16 kits/day). **Impact (new infections):** 11 of the 131 participants (8.4%) reporting a positive HIV test result. **Impact (first-time testers):** 63/254 (24.8%) participants had never tested for HIV before. **Impact (result return):** 131 out of the 177 participants (74%) who used an at-home self-test kit reported a self-test result. **Impact (linkage to care):** 9/11 (82%) reported that they sought confirmatory testing and 4 of these 9 (44.4%) had started treatment for HIV. Among the 120 participants who reported a negative test result for HIV infection, 13 (11%) reported visiting a provider to discuss PrEP or reported starting PrEP. |
| Thakker et al., 2022 [48] | **Feasibility (uptake):** 1356/2234 (61%) of registered clients ordered an HIVST. **Impact (new infections):** 43/1070 (4%) were HIV positive. **Impact (first-time testers):** 0/43 of those who tested positive had been tested for HIV before. **Impact (result return):** 1070/1190 (90%) of those who received their kit within 3 days uploaded their results. **Impact (linkage to care):** 19/43 (44%) of those that tested positive were linked to confirmatory testing, 16/19 (84%) of who were confirmed to be positive, and 14/16 (88%) started ART. |
| Tharao et al., 2022 [49] | **Impact (first-time testers):** 56/255 (22.0%) of participants were first-time testers. |
| Vasconcelos et al., 2022 [50] | **Feasibility (uptake):** 1356/2234 (61%) of registered clients ordered an HIVST. **Impact (new infections):** 43/1070 (4%) were HIV positive. **Impact (first-time testers):** 0/43 of those who tested positive had been tested for HIV before. **Impact (result return):** 1070/1190 (90%) of those who received their kit within 3 days uploaded their results. **Impact (linkage to care):** 19/43 (44%) of those that tested positive were linked to confirmatory testing, 16/19 (84%) of who were confirmed to be positive, and 14/16 (88%) started ART. |
| Vlasiuk et al., 2022 [51] | **Feasibility (uptake):** 21,035 HIVST kits were ordered.  **Impact (new infections):** 86 people who conducted the HIVST were confirmed to be positive at project sites. |
| Young et al., 2022 [52] | **Feasibility (uptake):** Greater proportion of those in the intervention group accepted the offer for an HIVST (29% - 130/450) compared to the control group (23% - 102/450) (OR=1.43, 95%CI, 1.04-1.95, p=0.027). **Feasibility (response rate):** 421/450 (93.4%) of the intervention group and 418/450 (92.9%) of the control group completed the follow-up survey. |
| Zhou et al., 2023 [53] | **Feasibility (uptake):** Rate of uptake increased from 68.0% to 87.2%. **Impact (new infections):** 1.0% (3/300) of participants reported a positive self-test result. |
| Zhou et al., 2022 [54] | **Feasibility (uptake):** 222 kits, 275 kits, and 337 kits were ordered from the control, SD-M, and SD-M-PR arms respectively. **Feasibility (response rate):** 96/102 (94.1%) control, 97/103 SD-M (94.2%), and 100/104 SD-M-PR (96.2%) participants did the follow-up survey. **Impact (new infections):** 18 people were diagnosed with HIV (15 newly diagnosed). **Impact (first-time testers):** 47/309 (15%) had never tested for HIV. **Impact (result return):** 209/222 (94.1%) results returned from control group, 257/275 (93.4%) results returned from SD-M group, and 328/337 (97.3%) results returned from SD-M-PR group. |
| Zhu et al., 2024 [55] | **Feasibility (uptake):** 92.9% (26/28) of participants performed the HIVST. **Feasibility (visits to web-based provider):** 67.9% (19/28) of participants clicked on the messages sent on the app. **Impact (result return):** 100.0% (26/26) of participants who conducted the self-test reported their result via photo. |

**References**

1. Beecroft AM, Duchaine T, Engel N, Liang C, Esmail A, Dheda K, et al. 1513. Evaluating the Incremental Accuracy of HIV Self-Test Together with an App-Based Solution: A Secondary Data Analysis of Trial Data. Open Forum Infect Dis. 2023 Dec;10(Suppl 2). doi: 10.1093/ofid/ofad500.1348.

2. Brady M, Carpenter G, Bard B. Self-testing for HIV: Initial experience of the UK's first kit. HIV Medicine. 2016;17(Supplement 1):9. doi: <https://dx.doi.org/10.1111/hiv.12392>.

3. Doan T, Stafylis C, Wang Q, Vavala G, Lemley S, McLeman B, et al. Accuracy of interpretation and home test kit result reporting for screening of human immunodeficiency virus infection. Sexually Transmitted Infections. 2021;97(SUPPL 1):A83. doi: <https://dx.doi.org/10.1136/sextrans-2021-sti.219>.

4. Kwan TH, Chan DPC, Wong SY, Lee SS. Implementation Cascade of a Social Network-Based HIV Self-testing Approach for Men Who Have Sex With Men: Cross-sectional Study. J Med Internet Res. 2023 Apr 26;25:e46514. PMID: 37099364. doi: 10.2196/46514.

5. O'Byrne P, Musten A, Orser L, Horvath C. Invalid Results in the GetaKit Study in Ottawa: A Real-World Observation of the INSTI HIV Self-test Among Persons At Risk for HIV. The Journal of the Association of Nurses in AIDS Care : JANAC. 2022;33(5):567-73. doi: <https://dx.doi.org/10.1097/JNC.0000000000000335>.

6. Roche SD, Ekwunife OI, Mendonca R, Kwach B, Omollo V, Zhang S, et al. Measuring the performance of computer vision artificial intelligence to interpret images of HIV self-testing results. Front Public Health. 2024;12:1334881. PMID: 38384878. doi: 10.3389/fpubh.2024.1334881.

7. Wang X, Tang Z, Wu Z, Nong Q, Li Y. Promoting oral HIV self-testing via the internet among men who have sex with men in China: a feasibility assessment. HIV Medicine. 2020;21(5):322-33. doi: <https://dx.doi.org/10.1111/hiv.12830>.

8. Bell SFE, Lemoire J, Debattista J, Redmond AM, Driver G, Durkin I, et al. Online HIV self-testing (HIVST) dissemination by an australian community peer HIV organisation: A scalable way to increase access to testing, particularly for suboptimal testers. International Journal of Environmental Research and Public Health. 2021;18(21):11252. doi: <https://dx.doi.org/10.3390/ijerph182111252>.

9. Birdthistle I, Mulwa S, Sarrassat S, Baker V, Khanyile D, O'Donnell D, et al. Effects of a multimedia campaign on HIV self-testing and PrEP outcomes among young people in South Africa: a mixed-methods impact evaluation of MTV Shuga Down South'. BMJ Global Health. 2022;7(4):e007641. doi: <https://dx.doi.org/10.1136/bmjgh-2021-007641>.

10. Chan PS, Chidgey A, Lau J, Ip M, Lau JTF, Wang Z. Effectiveness of a Novel HIV Self-Testing Service with Online Real-Time Counseling Support (HIVST-Online) in Increasing HIV Testing Rate and Repeated HIV Testing among Men Who Have Sex with Men in Hong Kong: Results of a Pilot Implementation Project. Int J Environ Res Public Health. 2021 Jan 15;18(2). PMID: 33467770. doi: 10.3390/ijerph18020729.

11. Fischer AE, Phatsoane M, Majam M, Shankland L, Abrahams M, Rhagnath N, et al. Uptake of the Ithaka mobile application in Johannesburg, South Africa, for human immunodeficiency virus self-testing result reporting. Southern African Journal of HIV Medicine. 2021;22(1):a1197. doi: <https://dx.doi.org/10.4102/SAJHIVMED.V22I1.1197>.

12. Girault P, Wong CM, Jittjang S, Fongkaew K, Cassell MM, Lertpiriyasuwat C, et al. Uptake of oral fluid-based HIV self-testing among men who have sex with men and transgender women in Thailand. PLoS ONE. 2021;16(8 August):e0256094. doi: <https://dx.doi.org/10.1371/journal.pone.0256094>.

13. Guadamuz TE, Samoh N, Lim SH, Jonas KJ, editors. A pilot feasibility study of HIV self-testing with online supervision among MSM who attend sexualized drug parties in Thailand. International AIDS Society; 2022; Montreal, Canada.

14. Howells W, Winter A, Baraitser P. A Comparison of Service User Uptake of Selftesting Versus Self-Sampling during England's National Hiv Testing Week 2023 by Demographic Group and Risk Profile. Sexually Transmitted Infections. 2023 June;99(Supplement 1):A42-A3. PMID: 642131108.

15. Jin X, Lu T, Gao Y, Mao X, Chu Z, Zhang J, et al., editors. Effect of a regular self-testing kits distribution intervention on HIV testing and sexual-behavioral outcomes among MSM in China: a stepped-wedge randomized controlled trial. International AIDS Society; 2021; Virtual.

16. Johnson MC, Chung R, Leung SYJ, Edelstein Z, Yuan Y, Flavin SM. Combating Stigma Through HIV Self-Testing: New York State's HIV Home Test Giveaway Program for Sexual Minorities. Journal of public health management and practice : JPHMP. 2022;28(2):174-83. doi: <https://dx.doi.org/10.1097/PHH.0000000000001138>.

17. Kaneko N, Sherriff N, Takaku M, Vera JH, Peralta C, Iwahashi K, et al. Increasing access to HIV testing for men who have sex with men in Japan using digital vending machine technology. Int J STD AIDS. 2022 Jun;33(7):680-6. PMID: 35502984. doi: 10.1177/09564624221094965.

18. Kaptchuk RP, Thakker J, Bell J, Okram S, Gopinath U, Mehta SH, et al. HIV self-testing in India: implementation and qualitative evaluation of a web-based programme with virtual counsellor support. J Int AIDS Soc. 2024 Jun;27(6):e26302. PMID: 38861458. doi: 10.1002/jia2.26302.

19. Larsson L, Chikwari CD, McHugh G, Koris A, Bandason T, Dauya E, et al. Feasibility and Usability of Mobile Technology to Assist HIV Self-Testing in Youth in Zimbabwe: A Mixed-Methods Study. J Adolesc Health. 2023 Sep;73(3):553-60. PMID: 37389521. doi: 10.1016/j.jadohealth.2023.05.011.

20. Li S, Zhang J, Mao X, Lu T, Gao Y, Zhang W, et al. Feasibility of indirect secondary distribution of HIV self-test kits via wechat among men who have sex with men: National cross-sectional study in China. Journal of Medical Internet Research. 2021;23(10):e28508. doi: <https://dx.doi.org/10.2196/28508>.

21. Logie CH, Okumu M, Berry I, Hakiza R, Baral SD, Musoke DK, et al. Findings from the Tushirikiane mobile health (mHealth) HIV self-testing pragmatic trial with refugee adolescents and youth living in informal settlements in Kampala, Uganda. J Int AIDS Soc. 2023 Oct;26(10):e26185. PMID: 37850816. doi: 10.1002/jia2.26185.

22. Ly A, Lo Hog Tian JM, Zubowski L, Maraj D, McBain K, Chan P, et al., editors. l'm Ready, Test: Leveraging a mobile health app and program to reduce barriers and facilitate access to HIV self-testing in Canada to reach the undiagnosed. Canadian Association for HIV Research; 2023; Quebec City, Canada.

23. MacGowan RJ, Chavez PR, Dana R, Hannah M, Raiford JL, Caldwell JA, et al. Efficacy of Internet Recruitment and HIV Self-Testing for Diagnosing HIV Infections Among Black and Hispanic/Latino MSM and Transgender Women in 11 US States, 2020-2021. J Acquir Immune Defic Syndr. 2024 Oct 1;97(2):133-41. PMID: 39250647. doi: 10.1097/qai.0000000000003476.

24. Maraj D, Ly A, Lo Hog Tian JM, Zubowski L, Watson JR, McBain K, et al., editors. l'm Ready, Talk: Implementation of a peer navigator program to facilitate HIV self- testing and linkage to care in Canada. Canadian Association for HIV Research; 2023; Quebec City, Canada.

25. Marley G, Fu G, Zhang Y, Li J, Tucker JD, Tang W, et al. Willingness of Chinese men who have sex with men to use smartphone-based electronic readers for HIV self-testing: Web-based cross-sectional study. Journal of Medical Internet Research. 2021;23(11):e26480. doi: <https://dx.doi.org/10.2196/26480>.

26. McFall AM, Thakker J, Loeb TA, Bell J, Singh A, Pollard R, et al. Virtual Support Improves Client Experiences with an Online Hiv Self-Testing Service. Topics in Antiviral Medicine. 2023 April;31(2):373. PMID: 641190637.

27. Mshweshwe-Pakela NT, Mabuto T, Shankland L, Fischer A, Tsukudu D, Hoffmann CJ. Digitally supported HIV self-testing increases facility-based HIV testing capacity in Ekurhuleni, South Africa. South Afr J HIV Med. 2022;23(1):1352. PMID: 35923609. doi: 10.4102/sajhivmed.v23i1.1352.

28. Nguyen VTT, Dunkley Y, Son VH, Choko AT, Huong PTT, Manh PD, et al. Investigating the effectiveness of web-based HIV self-test distribution and linkage to HIV treatment and PrEP among groups at elevated risk of HIV in Viet Nam provinces: a mixed-methods analysis of implementation from pilot to scale-up. J Int AIDS Soc. 2024 Jul;27 Suppl 1:e26264. PMID: 38965974. doi: 10.1002/jia2.26264.

29. Ni Y, Lu Y, Jing F, Wang Q, Xie Y, He X, et al. A Machine Learning Model for Identifying Sexual Health Influencers to Promote the Secondary Distribution of HIV Self-Testing Among Gay, Bisexual, and Other Men Who Have Sex With Men in China: Quasi-Experimental Study. JMIR Public Health Surveill. 2024 Apr 24;10:e50656. PMID: 38656769. doi: 10.2196/50656.

30. Ni Y, Lu Y, Zhou Y, Tang W. Using social media and social network to expand HIV self-testing in china. Sexually Transmitted Infections. 2021;97(SUPPL 1):A12. doi: <https://dx.doi.org/10.1136/sextrans-2021-sti.40>.

31. Ntinga X, Musiello F, Keter AK, Barnabas R, Heerden AV. The Feasibility and Acceptability of an mHealth Conversational Agent Designed to Support HIV Self-testing in South Africa: Cross-sectional Study. Journal of Medical Internet Research. 2022;24(12):e39816. doi: <https://dx.doi.org/10.2196/39816>.

32. O'Byrne P, Kroch A, Orser L, Ho N, Musten A, Haines M, et al. Targeted HIV Self-Testing Identifies Persons with Undiagnosed HIV and Active Engagement Links them to Care: The GetaKit Study. AIDS Behav. 2024 Jun;28(6):2015-22. PMID: 38526637. doi: 10.1007/s10461-024-04302-5.

33. O'Byrne P, Musten A, McCready L, Robinson R, Durrant G, Tigert J, et al. HIV self-testing enabled access to testing for Black persons: The GetaKit study. Research in nursing & health. 2023;46(2):236-41. doi: <https://dx.doi.org/10.1002/nur.22293>.

34. O'Byrne P, Musten A, Ho H, Lindsay J, Orser L, editors. The GetaKit Study: Bridging the gap in undiagnosed HIV infections using at-home HIV self-testing kits. Canadian Association for HIV Research; 2023; Quebec City, Canada.

35. O'Byrne P, Musten A, Vandyk A, Ho N, Orser L, Haines M, et al. HIV self-testing in Ottawa, Canada used by persons at risk for HIV: The GetaKit study. Can Commun Dis Rep. 2021 Oct 14;47(10):435-41. PMID: 34737676. doi: 10.14745/ccdr.v47i10a06.

36. O’Byrne P, Winkelmant S, Musten A, Inamdar G, Orser L, Grayson MO, et al., editors. Mailout HIV self-testing: overview of the GetaKit initiative in Ottawa, Canada. International AIDS Society; 2021; Virtual.

37. Oladele DA, Iwelunmor J, Gbajabiamila T, Obiezu-Umeh C, Okwuzu JO, Nwaozuru U, et al. An Unstructured Supplementary Service Data System to Verify HIV Self-Testing Among Nigerian Youths: Mixed Methods Analysis of Usability and Feasibility. JMIR Form Res. 2023 Sep 25;7:e44402. PMID: 37747780. doi: 10.2196/44402.

38. Pai N, Esmail A, Saha Chaudhuri P, Oelofse S, Pretorius M, Marathe G, et al. Impact of a personalised, digital, HIV self-testing app-based program on linkages and new infections in the township populations of South Africa. BMJ Global Health. 2021;6(9):e006032. doi: <https://dx.doi.org/10.1136/bmjgh-2021-006032>.

39. Phatsoane Gaven M, Quaife M, Majam M, Singh L, Rhagnath N, Wonderlik T, et al. HIV self-test reporting using mHealth platforms: A pilot study in Johannesburg, South Africa. Front Reprod Health. 2023;5:1073492. PMID: 36923466. doi: 10.3389/frph.2023.1073492.

40. Pollard R, Okram S, Thakker J, Singh A, Bell J, McFall A, et al., editors. Tailoring HIVST to local communities can improve uptake: lessons learned from a virtual HIV self-testing intervention in India. International AIDS Society; 2022; Montreal, Canada.

41. Ramos SR, Lardier DT, Jr., Bond KT, Boyd DT, O'Hare OM, Nelson LE, et al. Participatory Design of a Web-Based HIV Oral Self-Testing Infographic Experiment (HOTIE) for Emerging Adult Sexual Minority Men of Color: A Mixed Methods Randomized Control Trial. Int J Environ Res Public Health. 2021 Nov 12;18(22). PMID: 34831644. doi: 10.3390/ijerph182211881.

42. Rosadino JDT, Pagtakhan RG, Brines MT, Dinglasan JLG, Cruz DP, Corciega JOL, et al. Implementation of unassisted and community-based HIV Self-Testing (HIVST) during the COVID-19 pandemic among Men-who-have-sex-with-Men (MSM) and Transgender Women (TGW): A demonstration study in Metro Manila, Philippines. PLoS ONE. 2023;18(3 March):e0282644. doi: <https://dx.doi.org/10.1371/journal.pone.0282644>.

43. Rourke S, Owino M, Tharao W, Lachowski N, Duddy J, Ly A, et al., editors. Effectiveness of the I'm Ready Program to provide low barrier access across Canada to HIV self-testing to reach first-time testers. Canadian Association for HIV Research; 2023; Quebec City, Canada.

44. Shaikezhanov A, Gryazev D, Terlikbayeva T, Primbetova S, Wu E, Hunt H, et al., editors. Preliminary results of AmanBol: HIV self-testing program for MSM and transgender persons in Kazakhstan. International AIDS Society; 2021; Virtual.

45. Shrestha RK, Hecht J, Chesson HW. Analyzing the Costs and Impact of the TakeMeHome Program, a Public-Private Partnership to Deliver HIV Self-Test Kits in the United States. J Acquir Immune Defic Syndr. 2024 Feb 1;95(2):144-50. PMID: 37831623. doi: 10.1097/qai.0000000000003323.

46. Shrestha R, Altice FL, Khati A, Azwa I, Gautam K, Gupta S, et al. Clinic-Integrated Smartphone App (JomPrEP) to Improve Uptake of HIV Testing and Pre-exposure Prophylaxis Among Men Who Have Sex With Men in Malaysia: Mixed Methods Evaluation of Usability and Acceptability. JMIR mHealth and uHealth. 2023 16 Feb;11:e44468. PMID: 640342311.

47. Stafylis C, Vavala G, Wang Q, McLeman B, Lemley SM, Young SD, et al. Relative Effectiveness of Social Media, Dating Apps, and Information Search Sites in Promoting HIV Self-testing: Observational Cohort Study. JMIR Form Res. 2022 Sep 23;6(9):e35648. PMID: 36149729. doi: 10.2196/35648.

48. Thakker JH, Singh A, Pollard R, Bell J, McFall AM, Taduri M, et al. HIV SELF-TESTING UNCOVERS HIGH BURDEN of HIDDEN INFECTIONS in INDIA. Topics in Antiviral Medicine. 2022;30(1 SUPPL):55.

49. Tharao W, Owino M, Aden M, Soje L, Keen S, Frans D, et al., editors. I'm Ready Program 6-Month Results: African, Caribbean and Black participants accessing HIV self-testing across Canada. Canadian Association for HIV Research; 2022; Virtual.

50. Vasconcelos R, Avelino-Silva VI, de Paula IA, Jamal L, Gianna MC, Santos F, et al. HIV self-test: a tool to expand test uptake among men who have sex with men who have never been tested for HIV in Sao Paulo, Brazil. HIV Medicine. 2022;23(5):451-6. doi: <https://dx.doi.org/10.1111/hiv.13178>.

51. Vlasiuk O, Shevchuk D, Gavrysh L, editors. Introduction of oral-based HIV self-testing in Ukraine through the national-scale HealthLink Project: distribution strategies and key results. International AIDS Society; 2022; Montreal, Canada.

52. Young SD, Cumberland WG, Singh P, Coates T. A Peer-Led Online Community to Increase HIV Self-Testing Among African American and Latinx MSM: A Randomized Controlled Trial. Journal of Acquired Immune Deficiency Syndromes. 2022;90(1):20-6. doi: <https://dx.doi.org/10.1097/QAI.0000000000002919>.

53. Zhou H, Zhu YY, Gao YY, Chu ZX, Chen S, Liu M, et al. Online distribution of HIV self-testing kits to promote HIV testing among men who have sex with men discontinuing pre-exposure prophylaxis after demonstration project completion in China: a multicentre open-label randomized controlled trial. Lancet Reg Health West Pac. 2023 Dec;41:100922. PMID: 37867621. doi: 10.1016/j.lanwpc.2023.100922.

54. Zhou Y, Lu Y, Ni Y, Wu D, He X, Ong JJ, et al. Monetary incentives and peer referral in promoting secondary distribution of HIV self-testing among men who have sex with men in China: A randomized controlled trial. PLoS Medicine. 2022;19(2):e1003928. doi: <https://dx.doi.org/10.1371/journal.pmed.1003928>.

55. Zhu Z, Lu X, Gao P, Wang X, Hu X, Xie N, et al. Feasibility of a Mobile Health Intervention for Providing a Continuum of HIV Services for MSM: Pilot Study of the WeTest Program in 3 Cities in China. Current HIV Research. 2024;22(3):158-69. PMID: 2030390952.
